# Motifs enable communication efficiency and fault-tolerance in transcriptional networks

**DOI:** 10.1038/s41598-020-66573-x

**Published:** 2020-06-15

**Authors:** Satyaki Roy, Preetam Ghosh, Dipak Barua, Sajal K. Das

**Affiliations:** 1 Department of Computer Science, Missouri University of Science and Technology, Missouri, USA; 2 Department of Computer Science, Virginia Commonwealth University, Richmond, USA; 3 Department of Chemical Engineering, Missouri University of Science and Technology, Missouri, USA; 4 University of North Carolina, Chapel Hill, USA

**Keywords:** Gene regulatory networks, Network topology

## Abstract

Analysis of the topology of transcriptional regulatory networks (TRNs) is an effective way to study the regulatory interactions between the transcription factors (TFs) and the target genes. TRNs are characterized by the abundance of motifs such as feed forward loops (FFLs), which contribute to their structural and functional properties. In this paper, we focus on the role of motifs (specifically, FFLs) in signal propagation in TRNs and the organization of the TRN topology with FFLs as building blocks. To this end, we classify nodes participating in FFLs (termed *motif central nodes*) into three distinct roles (namely, roles *A*, *B* and *C*), and contrast them with TRN nodes having high connectivity on the basis of their potential for information dissemination, using metrics such as network efficiency, path enumeration, epidemic models and standard graph centrality measures. We also present the notion of a three tier architecture and how it can help study the structural properties of TRN based on connectivity and clustering tendency of motif central nodes. Finally, we motivate the potential implication of the structural properties of motif centrality in design of efficient protocols of information routing in communication networks as well as their functional properties in global regulation and stress response to study specific disease conditions and identification of drug targets.

## Introduction

Transcriptional regulatory networks (TRNs) are biological networks that have attracted great interest in the field of systems biology. TRNs represent the regulation of genes as a function of the input signals received by proteins called *transcription factors* (TFs)^1,2^. It is believed that the understanding of the organizational principles of TRNs may contribute towards explaining its network architecture as well as the role of TFs in gene expression and maintenance of complex cellular processes^3^.

Network motifs are regulatory substructures in a network topology that appear more frequently than those in randomized networks^4–6^. Milo *et al*. reported the presence of motifs in biological networks (e.g., TRNs), ecological networks (e.g., food web), neurobiology (e.g., neuron connectivity maps) and engineering (e.g., electronic circuits, World Wide Web)^7^. Also, analysis of network motifs revealed key patterns of interconnections among users and patterns in social behavior in colored temporal social networks^8,9^. Clearly, the ubiquity of motifs in different kinds of complex networks makes their analysis and enumeration essential for the identification of structural design principles and key network interactions^10–12^.

Prior studies on the TRN topologies of *Escherichia coli* (*E*. *coli*) and *Saccharomyces cerevisiae* (*S*. *cerevisiae*) showed that a three-node motif, called feed forward loop (FFL), plays the roles of filters, pulse generators, response accelerators, temporal pattern generators and information-processing modules for robust circuits^13,14^, whereas a four-node motif, called bi fan, participates in nutrient metabolism and bio-synthesis^13,15^. *Considering the statistical significance of motifs*, *Milo et al*. *raised the question whether motifs have definite information*-*processing roles in complex networks such as TRNs*^7^. There are initial works that attempt to address this question. Kosyfaki *et al*. posited the notion of network flow motifs, a novel motif type that models information flow transfer among a set of vertices within a constrained time window^16^. Martens *et al*. showed that a bi-directional two-hop path is a motif that enables information flow in functional brain networks^17^. *However*, *there have been no efforts to study motifs through models (such as epidemic models) that may capture their information spread dynamics*. Another area of interest has been the organizational structure of complex networks with respect to network motifs. Kashtan *et al*. presented a systematic approach, called motif generalization, for uniting related groups of motifs into families. These generalizations, produced by replicating nodes in a basic motif structure, were shown to preserve the dynamical function of the motifs on which they are based^18^. Benson *et al*. presented a framework to identify clusters of network motifs that help reveal organizational patterns within complex systems^19^. Gorochowski *et al*. attempted to discover the nature of intrinsic aggregation among smaller (such as three-node) motifs that constitute larger substructures^20^. *How the nature of connectivity between motifs impacts information dissemination (by means of diffusion as defined in Sec*. *2*.*2*.*2)* in the network continues to be explored.

**Figure 1:**
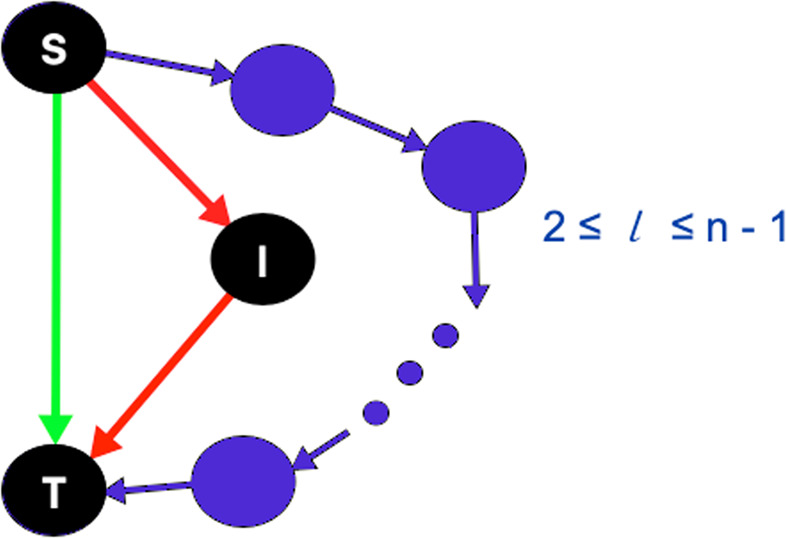
The directed edge $(S,I)$ allows unidirectional information flow from node $S$ to $I$; feed forward loop (FFL) motif: the source $S$ is the master regulator, $I$ is intermediate regulator and target $T$ is called the regulated vertex. Given any simple n-node directed graph, if the directed link from node $S$ to $T$ (marked green) is removed, $T$ may often become unreachable from $S$, or the length of the indirect shortest path length (denoted by $l$) may range between 2 and $n-1$. Due to the presence of an FFL, if the direct link between $S$ and $T$ marked in green is removed, the indirect path (via *I*) shown in red has length $l=2$.

There exist 13 possible three-node motifs (or non-isomorphic connected directed triads)^21^; out of these the feed forward loop (FFL) motif (defined below and depicted in Fig. 1) is not only observed abundantly in the TRNs of both *E*. *coli* and *S*. *cerevisiae*^7^ outnumbering the other three-node cyclic triangle called feedback loop (see Appendix A of Supplementary Materials), but it is also a well-studied complex network motif. For instance, Abdelzaher *et al*. introduced a motif-based preferential attachment technique that yields network topologies that compare well with the *E*. *coli* TRN in terms of abundance of FFLs^15,22^. Furthermore, they showed that FFL abundance in TRNs is correlated with low average shortest path length and high clustering coefficients^23^. In an attempt to study the role of motifs in signal processing, Mangan *et al*. showed that two FFL structure types (termed coherent and incoherent FFLs) cause sign-sensitive delay and acceleration to incoming signal^14^. Gorochowski focused on the organization of FFLs into clusters in natural and engineered networks^20^. Abdelzaher *et al*. showed that FFL abundance in TRNs is correlated with low average shortest path length and high clustering coefficients^23^. *Based on these studies*, *we infer that characterization of the connectivity among FFLs at a node level (i*.*e*., *based on the FFL participation of nodes) may help understand the topological and functional roles of motifs in TRN and*, *by extension*, *in any complex network*.

*Feed forward loop (FFL) motif* consists of TF *S*, which regulates the second TF *I* (Fig. 1). $S$ and $I$ bind the regulatory region of the target gene $T$ and regulate its transcription^7,14^. Kashtan *et al*. referred the three-nodes in an FFL based on their functions^18^. Node $S$ is the master TF, $I$ is the intermediate regulator TF and $T$ is called the regulated vertex. Figure 1 depicts that FFL has two distinct paths from $S$ to $T$: the *direct edge* $S\to T$ (marked in green) and the *indirect path* $S\to I\to T$ (marked in red).

We hypothesize that FFL motifs provide robust pathways for information propagation in TRN based on the following reasons:

1. *Robustness due to independent paths:* Two paths between a node pair are called *independent* if they contain no common nodes, except source and destination nodes. According to Menger’s theorem on vertex connectivity, the minimum number of vertices whose removal disconnects two nodes is equal to the maximum number of pairwise vertex-independent paths between them^24^. In other words, since the FFL motif contains two independent paths connecting nodes $S$ and $T$, abundance of FFLs ensures topological robustness in TRNs by offering multiple alternative communication pathways. With this intuition, we have earlier explored the role of motifs in topological robustness of *E*. *coli* and *S*.*cerevisiae* TRNs; here we showed that FFL motifs render robustness to TRNs by creating multiple independent communication pathways, which may be utilized to design of fault-tolerant and energy-efficient dynamic communication network topologies^25,26^.
2. *Least increase in shortest path length:* Consider a simple *n*– node directed graph. (A simple graph has no parallel edges between a vertex pair or self-loops, as formally defined in Sec. 2.1). There are two possible consequences of knocking off the direct link from any node $u$ to $w$: (a) $w$ becomes unreachable from $u$, or (b) the shortest path length $l$ (where $2\le l\le n-1$) from $u$ to $w$ increases, which commensurately hampers how quickly information propagates from the source to the destination node. (Note that the shortest path length problem is about finding a path between vertices in a graph such that the total sum of the edge weights is minimum. In an unweighted directed graph, it is the path with the least number of edges between the node pair). Figure 1 shows that the presence of FFL motif ensures that the failure of direct link between source $S$ and target $T$ causes the least possible increase in shortest path (through intermediary $I$ marked in red) length (i.e, $l=2$)^25^. Therefore, we hypothesize that cascades of FFLs should make TRNs resilient by minimizing the increase in shortest path length due to node and link failures.
3. *FFLs as statistically significant subgraphs in TRNs:* FFL motifs have been shown to be the “building blocks”, i.e., they are over-represented subgraphs in biological networks like TRNs^20,27,28^. We used a motif detection tool, called FANMOD^11^ to show that some of the abundant 4–, 5– and 6–node motifs contain FFL motifs (see Appendix B of Supplementary Materials). We intuit that the information flow in TRNs can be effectively analyzed w.r.t. the FFL motifs.

*Our contributions*: So far we highlight the following specific unanswered questions pertaining to motifs: (a) what is their role in information propagation in TRNs and (b) how are they organized as building blocks leading to the formation of the TRN topology? We seek answers to these questions through experiments considering TRN nodes, called *motif central nodes*, with high FFL motif participation. We formally define three types of motif central nodes (i.e., roles *A*, *B* and *C*) and study their impact on the topological and functional property in TRN using existing network science as well as biological metrics. Specifically, with regard to *information dissemination* (defined in Sec. 2.2.2), we employ graph centrality and epidemiological models to analyze the extent to which motif central nodes participate in information diffusion, and quantify *fault*-*tolerance* (defined in Sec. 2.2.3) by studying the effect that removal of motif central nodes have on network efficiency. Moreover, we utilize a three tier topological characterization to gain insights into the organization of FFLs in TRNs as well as their mutual connectivity. Finally, we analyze the potential overlap between the topological role of FFLs and their functional role as *master regulators* (see Sec. 2.2.1) and in stress response, before discussing their implications in the design of protocols for routing information across communication networks as well as identification of drug targets in the field of disease biology (see Sec. 4).

## Materials and Methods

**Figure 2:**
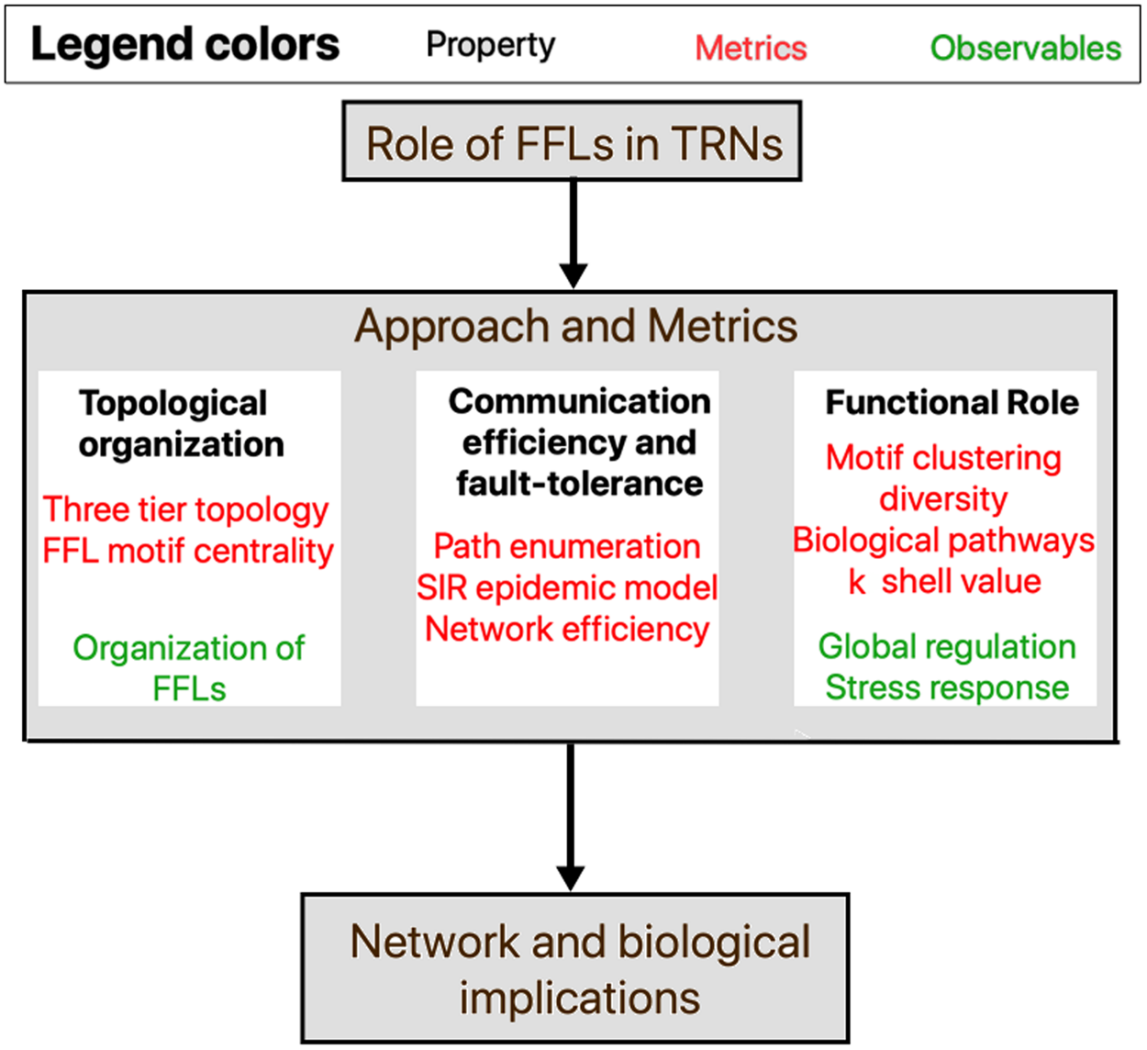
Contributions of the paper: Role of the feed forward loop (FFL) motifs in TRN in terms of three properties (shown in bold black) with the associated network science based metrics (colored red), leading to an analysis on how the observables (colored green) can motivate future research in communication network protocol design and drug target identification.

We define the graph-theoretic notions of directed graph, degree, path and graph density in Sec. 2.1. We then analyze the TRNs in relation to the following properties: (1) topology organization w.r.t. master regulators and FFL motif centrality (Sec. 2.2.1), (2) information dissemination and communication efficiency (Sec. 2.2.2), (3) fault-tolerance (Sec. 2.2.3) and (4) functional role (Sec. 2.2.4). Figure 2 depicts how we apply network science based metrics (colored red) within the different roles or properties of FFL motifs (shown in bold black) in TRN; in the final step, we study the implications of the observables (marked green) in the design of communication network routing protocols as well as drug targets identification for disease scenarios.

### Preliminaries

#### Directed graph

A graph is an ordered pair $G=(V,E)$ where *V* is a finite, non-empty set of objects called *vertices* (or nodes); and *E* is a (possibly empty) set of 2-subsets of *V*, called *edges*^29^. A directed graph is a graph where edges have directions. A directed edge $(S,T)\in E$ allows unidirectional information flow from vertex $S$ to $T$ and not necessarily from $T$ to $S$ (see Fig. 1)^30^. Contrast this with an undirected graph where $(S,T)$ has no direction allowing two-way information flow between $S$ and $T$.

#### Degree, path and path length in directed graph

The number of edges leaving a node $u$ is termed its *out*-*degree* (denoted by $de{g}^{+}(u)$) and number of edges entering a node is its *in*-*degree* (denoted by $de{g}^{-}(u)$). A *directed path* is a sequence of vertices such that there is a directed edge pointing from each vertex to its successor in the sequence, with no repeated edges. We represent a path as $p=\{{u}_{j},{u}_{j+1},\cdots ,{u}_{n}\}$, where $({u}_{i},{u}_{i+1})\in E$ ($j\le i < n$). A directed path is considered *simple*, if it has no repeated node, except the starting and ending node. The length of a simple path is calculated as the number of edges it contains. Any given node $v$ is considered to be *unreachable* from $u$, if there exists no directed path from $u$ to $v$ in *G*.

#### Graph density

It is the ratio between the number of edges and the maximum possible number of edges in a graph. For a directed graph *G*(*V*, *E*), it is measured as:

1 $$D=\frac{|E|}{|V|\times (|V|-1)}$$

An empty graph has density $D=0$, while a complete graph, having all possible directed edge connections, has density $D=1.0$.

#### Transcriptional regulatory networks

TRNs are represented as directed, signed graphs in which nodes represent genes or transcription factors (TFs) and edges correspond to the regulations of target genes by TFs^31^. Directed edges of TRNs are assigned positive or negative signs, indicating that the TF respectively increases or decreases the rate of transcription when it binds the promoter of the gene^32–34^.

#### Datasets

The validated and nearly complete TRNs of *Escherichia coli* (*E*. *coli*) and *Saccharomyces cerevisiae* (*S*.*cerevisiae*) were extracted from GeneNetWeaver^34^. This tool generates topologies by extracting modules based on biological interactions in *E*. *coli* and *S*.*cerevisiae*. *Homo sapiens* (human) and *Mus musculus* (mouse) TRNs were obtained from the TRRUST database^35^; these two TRNs catalogue the partially known validated interactions between TFs and genes in these organisms. The orders, sizes and graph densities (showing the sparse nature) of the four TRN topologies considered are summarized in Table 1.

**Table 1:** TRN graphs and corresponding graph densities (*D*).

TRN type	E. coli	S.cerevisiae	Human	Mouse
No. of nodes	$1565$	$4441$	$2862$	$2456$
No. of edges	$3758$	$12873$	$8427$	$6490$
D	$0.00150$	$0.00065$	$0.00102$	$0.00107$

Note that the complete information of the sign (i.e. up or down regulation) and magnitude of influence of TFs on their target genes (measured in terms of their expression values and corresponding rate constants) is not available in all these datasets. Therefore, in this paper we consider TRNs as unweighted, unsigned and directed graphs.

### Approach and metrics

We study the role of FFL motifs in TRNs from four different standpoints, namely, *topological organization of TRNs with respect to FFLs*, *communication efficiency*, *fault*-*tolerance* and *functional role*. We specifically define (and depict in Fig. 2) each of these four properties of FFLs in TRN and the associated metrics that quantify them.

#### Topological organization

We analyze the *topological structure of TRNs with respect to FFL motifs* to identify their logical communication architecture, comprising individual FFL units. To this end, we employ two node-level measures: (1) a characterization of TRN nodes into three tiers based on degree distribution and (2) participation of TRN nodes in FFLs. Based on these metrics, we analyze the distribution and organization of FFL motifs as well as the connectivity and distance among them within the TRN topology.

1. *Three tier topology:* It is the logical characterization of each TRN node into one of three tiers^36–38^ based on its in- and out-degree (as depicted in Fig. 3(a)) as follows:
  **Tier 1** (${T}_{1}$) consists of the set of nodes with only out-degree edges (i.e., $\{u\in V:de{g}^{-}(u)=0\}$). These nodes constitute approximately 5% of the total TRN nodes and are TFs.
  **Tier 2** (${T}_{2}$) consists of the set of nodes with non-zero in and out-degree edges (i.e., $\{u\in V:de{g}^{+}(u) > 0\,and\,de{g}^{-}(u) > 0\}$). These nodes also constitute approximately 5% of the total TRN nodes and are also TFs.
  **Tier 3** (${T}_{3}$) comprises the set of nodes with only in-degree edges (i.e., $\{u\in V:de{g}^{+}(u)=0\}$). These nodes are the genes and constitute the remaining 90% of the total TRN nodes.

**Figure 3:**
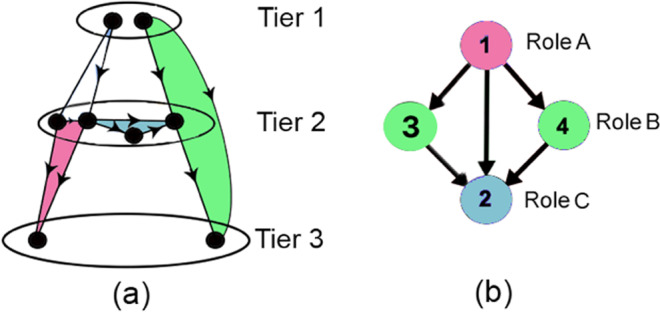
Topological characterization and FFL motif centrality in TRN: (**a**) Three tier TRN architecture taken from^37^ (each tier shown by ${T}_{i}$ for $i=1,2,3$). FFLs located across tiers ${T}_{1}\to {T}_{2}\to {T}_{2}$, ${T}_{1}\to {T}_{2}\to {T}_{3}$, ${T}_{2}\to {T}_{2}\to {T}_{2}$, ${T}_{2}\to {T}_{2}\to {T}_{3}$ denoted by white, green, blue and magenta, (**b**) An example subgraph with two FFL motifs consisting nodes $(1,4,2)$ and $(1,3,2)$, to depict the three roles in FFL motif centrality. Node 1 (colored magenta) and node 2 (colored blue) play roles *A* and *C* in both motifs, but nodes 3 and 4 (colored green) plays role *B* in motifs $(1,3,2)$ and $(1,4,2)$, respectively.
  The ${T}_{3}$ nodes are called *non*-*hubs* and the high out-degree nodes in ${T}_{1}$ and ${T}_{2}$ are called *hubs*; many of these hubs are *master regulators* (MRs), which control the expression of several other TFs and genes^39^. The high connectivity of these MRs allow them to participate in many FFL and FBL motifs and are studied to identify biomarkers for specific disease conditions, as discussed in Sec. 2.2.4^40^. Note that considerable effort has already gone into the analysis of the hierarchical structure of TRNs. Gerstein *et al*. studied the network interactions of different TFs and mRNAs in humans on the basis of properties such as hubs vs. non-hubs, connectivity, motifs, etc.^41^. Similarly, Bhardwaj *et al*. employed breadth-first search (BFS) to form a hierarchy of TFs based on regulating-regulated TF relationships to identify the master regulators in *E*. *coli* and *S*.*cerevisiae* TRNs^42^. Finally, the five-level hierarchy of TFs and operons in *E*. *coli* proposed by Ma *et al*.^43^ is the closest to our proposed three tier topology, which was simply intended to capture how hub nodes are responsible for the network connectivity as well as the unidirectional information flow from the hubs to the non-hubs of TRN^37^.
2. *FFL motif centrality*: It is a measure of the number of FFL motifs a node participates in. We subsequently formalize this notion (in Eq. 3) in terms of an indicator variable **M**, which defines the existence of a FFL motif between any ordered triplet of nodes $u,v,w\in V$ based on the presence of edges $(u,v),(v,w),(u,w)\in E$, as follows:

2 $${\bf{M}}(u,v,w)=\{\begin{array}{ll}1, & {\rm{if}}\,(u,v),(v,w),(u,w)\in E\\ 0, & {\rm{otherwise}}\end{array}$$

For instance, in Fig. 3(b), ${\bf{M}}(1,3,2)=1$, whereas ${\bf{M}}(1,3,4)=0$.

##### Roles of nodes in motif centrality

Recall from our discussion in Sec. 1 that if $M(u,v,w)=1$, *u* is the master regulator, $v$ is the intermediate regulator and $w$ is the regulated entity. Koschützki coined the names roles *A*, *B* and *C* to the three respective nodes participating in a FFL motif^44^. Specifically, for any given node $u$,

- *role A motif centrality* is the number of FFLs where node $u$ is the master TF, i.e., ${\delta }_{A}(u)=|\{{\bf{M}}(u,v,w):u,v,w\in V\}|$
- *role B motif centrality* is the number of FFLs where $u$ is the intermediate regulator TF, i.e., ${\delta }_{B}(u)=|\{{\bf{M}}(v,u,w):u,v,w\in V\}|$
- *Role C motif centrality* is the number of FFLs where $u$ is the regulated node, i.e., ${\delta }_{C}(u)=|\{{\bf{M}}(v,w,u):u,v,w\in V\}|$

Finally, the *FFL motif centrality* of any node $u\in V$ is calculated as:

3 $$\delta (u)={\delta }_{A}(u)+{\delta }_{B}(u)+{\delta }_{C}(u).$$

For example, in FFL motif $(1,4,2)$ *of* Fig. 3(b), nodes 1, 4 and 2, marked magenta, green and blue, respectively, play roles *A*, *B* and *C*; similarly, in the adjoining FFL $(1,3,2)$ nodes 1, 3 and 2 play roles *A*, *B* and *C*. For node 1, ${\delta }_{A}=2$, ${\delta }_{B}=0$ and ${\delta }_{C}=0$, respectively. Thus, by Eq. 3, $\delta (1)=2$ indicates that node 1 participates in 2 FFLs.

##### Direct link motif centrality list

For any edge $(u,w)$, direct link motif centrality list is defined as the list of nodes ${v}_{i}$ such that ${v}_{i}$ is the intermediate regulator in a FFL motif where $u$ and $w$ are direct regulators and regulated nodes respectively, i.e., ${\phi }_{d}((u,w))=\{{v}_{i}:{\bf{M}}(u,{v}_{i},w)=1,{v}_{i}\in V\}$. In Fig. 3(b), ${\phi }_{d}((1,2))=\{3,4\}$, since nodes 3 and 4 are intermediate regulators in FFL motifs $(1,3,2)$ and $(1,4,2)$, where $(1,2)$ forms a direct link.

The three tier topological characterization, coupled with FFL motif centrality, not only helps understand the TRN topology with FFLs as building blocks, but also contrast nodes with high motif centrality (termed *motif central nodes*) from those with high out-degree (termed *hubs*), on the grounds of communication efficiency and fault-tolerance (defined in Sec. 2.2.2 and 2.2.3).

#### Communication efficiency

*Dissemination of information* in a network follows *diffusion* wherein a node in possession of information transfers it to other nodes across its outgoing edges^24^. The process of information dissemination is similar to the flow of fluid in a network of pipes, which takes place simultaneously along multiple ‘fronts’ and not towards a specific destination^45^. *Communication efficiency* is a measure of how rapidly a node/motif can disseminate information to as many number of nodes in the network as possible. Specifically, we measure the communication efficiency rendered by FFL motifs in TRNs (1) in terms of the number of directed paths created as a result of the direct link and indirect path present in each FFL and (2) using the Susceptible-Infected-Recovered (SIR) epidemic model to gauge the diffusion of information (in terms of *infection* in epidemiology, or simply protein/mRNA molecules in signalling or regulatory networks) across each TRN over time when FFL motifs are activated as initial carriers of the infection. Note that the use of the SIR epidemic model is a common practice in studies on information spread in social networks^46^ and biological networks^47^. We also use centrality metrics such as closeness, betweenness and degree centralities to corroborate our findings on information spreading potential of FFL motifs. Refer to^48^ for details on these centrality measures.

1. *Path enumeration*: We utilize the notion of *direct link motif centrality* (discussed under *FFL motif centrality* in Sec. 2.2.1) to propose a simple heuristic to understand the extent to which the direct and indirect links of FFL motifs (discussed in details in Sec. 1) are responsible for creating simple paths from tier 1 to tier 3 nodes in TRN. The details of the working of the heuristic and an illustrative example has been shown in Appendix C of Supplementary Materials.
2. *Spread of infection modeled using the Susceptible Infected Recovered (SIR) model*: The SIR model is an epidemiological model to represent the contagion of an infectious disease in a large population. In this model, the total population is divided into three disjoint groups, namely, susceptible, infected and recovered. The infection rate (*β*) defines the probability of a infected node passing its infection to a neighboring susceptible node through diffusion, while recovery rate (*γ*) is the probability of any infected node attaining immunity from future infections.

##### Approach

We infect a small fraction of the initial population in *E*. *coli*, *S*. *Cerevisiae*, human and mouse TRN and record the spread of infection (i.e., the number of infected nodes) over a period of time. We consider two distinct scenarios of infection rate *β*/*γ*: (a) $\frac{\beta }{\gamma } < 1$ and (b) $\frac{\beta }{\gamma } > 1$. We employ the python SimPy library^49^ to simulate SIR epidemic on each TRN topology; each experiment has been carried out $100$ times. As mentioned before, we employ three standard graph centrality measures (namely, *degree*, *closeness* and *betweenness* centralities) to corroborate our findings.

#### Fault-tolerance

Fault-tolerance of a network is its ability to maintain *connectivity* despite the failure of components (such as a set of nodes)^50^. There exists several metrics (such as algebraic connectivity, effective resistance, and average edge betweenness) to estimate fault-tolerance, also called robustness, of a network^51^; we quantify connectivity (or the lack of it) of TRN in terms of a widely used metric called network efficiency, which measures how effectively the network exchanges information. Specifically, *network efficiency* is defined as the harmonic mean of shortest path lengths between all node pairs^52,53^. It is calculated as:

4 $$Z=\frac{1}{|V|\times (|V|-1)}\,\sum _{u,v\in V}\,\frac{1}{d(u,v)}$$

Here, *d*(*u*, *v*) denotes the shortest path length between nodes *u* and *v*. Note that the *unreachability* of *v* from *u* (discussed in Sec. 2.1) is represented as $d(u,v)=\infty$, and $1/d(u,v)=0$ in accordance with the concept of limits, ${\mathrm{lim}}_{x\to \infty }\frac{1}{x}=0$.

From the formulation in Eq. 4, one may infer that the network efficiency $Z$ is higher if the shortest path length between any given pair of nodes $u,v\in V$ is lower and information propagates quickly from $u$ to $v$. Also, note that a directed network is considered well-connected if most nodes are reachable from one another. Given that $Z$ is low when a high number of nodes are unreachable from one another, this metric also captures the extent of network connectivity. Thus, a fault-tolerant network, which, by definition, is able to maintain connectivity despite the removal of certain nodes, exhibits a high $Z$. In fact, network efficiency has been utilized to study disruption in information propagation in functional brain networks^54,55^. Here, we quantify effect of a specific set of nodes on the fault-tolerance of a network by recording the drop in $Z$ when such nodes are knocked off.

*Communication efficiency* and *fault*-*tolerance* are interrelated notions, since the networks exhibiting fault-tolerance are typically the ones that maintain a steady communication efficiency despite node failures. We therefore combine the result for the two stated aspects in Sec. 3.2. It is noteworthy that our earlier works on bio-inspired networking enabled the wireless networks to mimic TRN topologies for routing data packets, demonstrating significantly high communication efficiency and fault tolerance despite the failure of random nodes and edges^56–58^, leading to an indirect quantification of two measures.

#### Functional role of motif central nodes

Given that FFL motif central nodes play a role in communication efficiency and fault-tolerance of TRNs, we explore their functional roles as per published literature. As shown in Fig. 2, we utilize the following three metrics to pinpoint the functional role of motif central nodes. The first metric, *motif clustering diversity* (MCD), quantifies the participation of any given node in unique FFL clusters in terms of the number of different motif clustering types (called *configurations*) that the node takes part in. Its value ranges between 0 and 12. Gorochowski *et al*. showed that several nodes exhibiting high MCD act as global regulators controlling the transcription of several genes^20^. The second metric is *participation of a TF*/*gene in biological pathways*, which demonstrates its role in signal transduction pathways, gene regulation and metabolism. The third metric is the *k shell decomposition*, which reveals whether a node lies in the core or periphery of a network. Node with high k values (i.e. nodes lying in the network cores) have been shown to be the most efficient information spreaders in the networks^59^. We study whether TFs with high k values are also master regulators. (The details of the calculation of the three metrics have been discussed in Appendix D of Supplementary Materials). Finally, we validate our intuition that different classes of motif central nodes serve the function of regulators (from a communication efficiency angle) and cellular stress response (from a fault tolerance perspective).

We collate our findings on the roles of FFLs (defined in Sec. 2.2.1–2.2.4) to draw inferences on the potential *network and biological implications* of FFLs in TRNs. We first present a hub-and-spoke representation of the TRNs comprising motif central nodes that can motivate the design of new and efficient communication network routing protocols. Furthermore, given that several high NMC TFs/genes participate in global regulation and stress response, we examine whether FFL motif centrality may be used as an independent biomarker for understanding specific disease conditions and identification of drug targets.

## Results

### Topological organization of TRN w.r.t FFLs

In this section, we investigate the *distribution*, *connectivity* and *clustering* among FFL motifs in terms of the role $A$, $B$ and $C$ motif central nodes across the tiers (introduced in Sec. 2.2.1) of *E*. *coli*, *S*. *cerevisiae*, human and mouse TRNs. To achieve this, *we define high motif central nodes as nodes with total FFL motif centrality (*δ*) greater than or equal to* 100*; although this cut*-*off is arbitrary*, *it roughly accounted for a small fraction (*~1–2%*) of the TRN nodes*. In the rest of the manuscript, we utilize the abbreviation NMC to denote the total FFL motif centrality (i.e. *δ* defined in Eq. 3).

#### Distribution and connectivity of the motif central nodes

**Figure 4:**
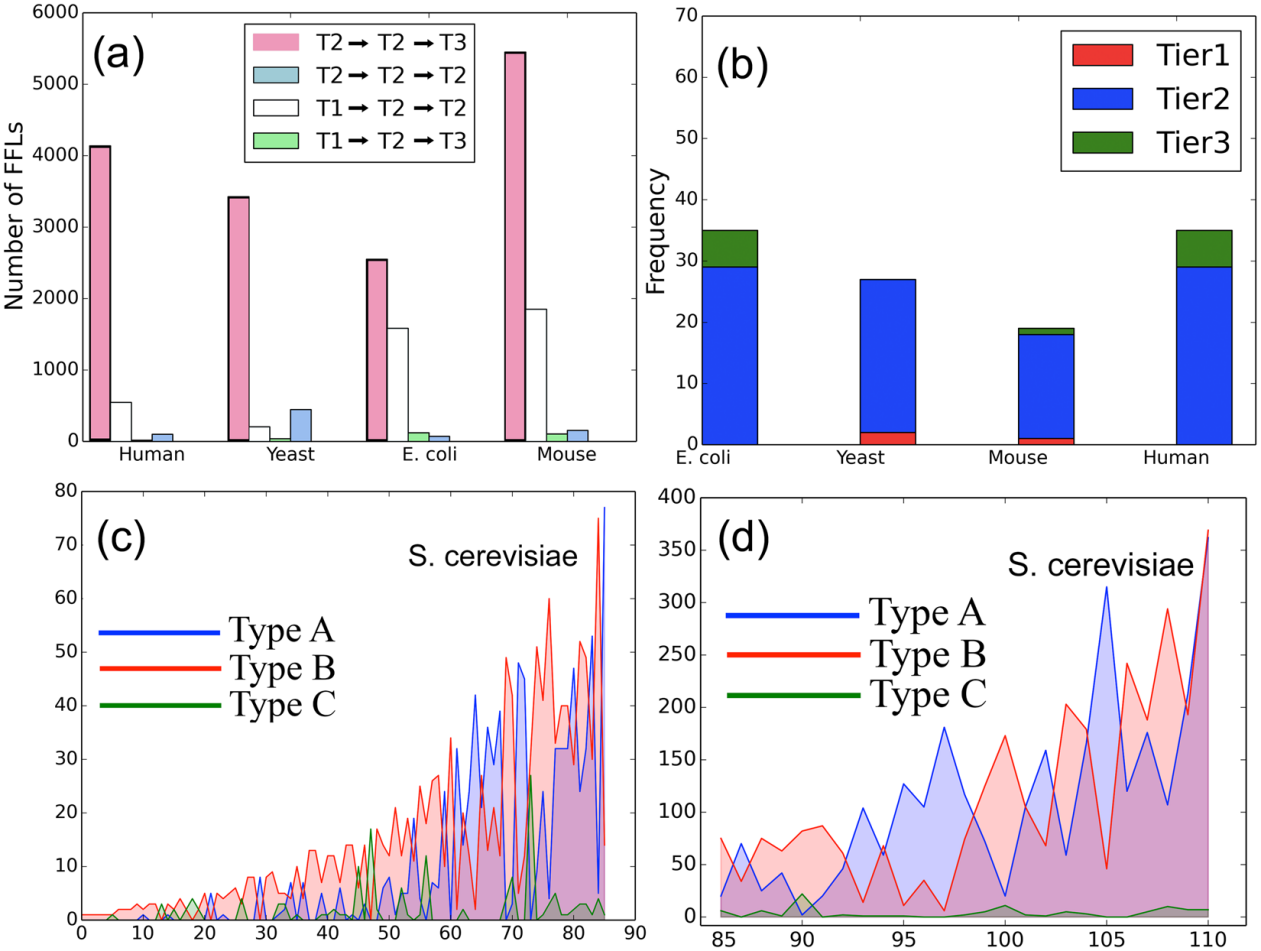
Distribution and connectivity among motif central nodes: (**a**) Spread of FFL motifs across the three tiers, (**b**) The classification of high NMC nodes across tiers, and (**c**,**d**) Role *A*, *B* and *C* participation of nodes with FFL motif centrality less than 100 and greater than 100, respectively (X axis: nodes in tier 2 arranged in the non-decreasing order of motif centrality; Y-axis: the frequency of role *A*, role *B* and role *C* motif centrality).

**Figure 5:**
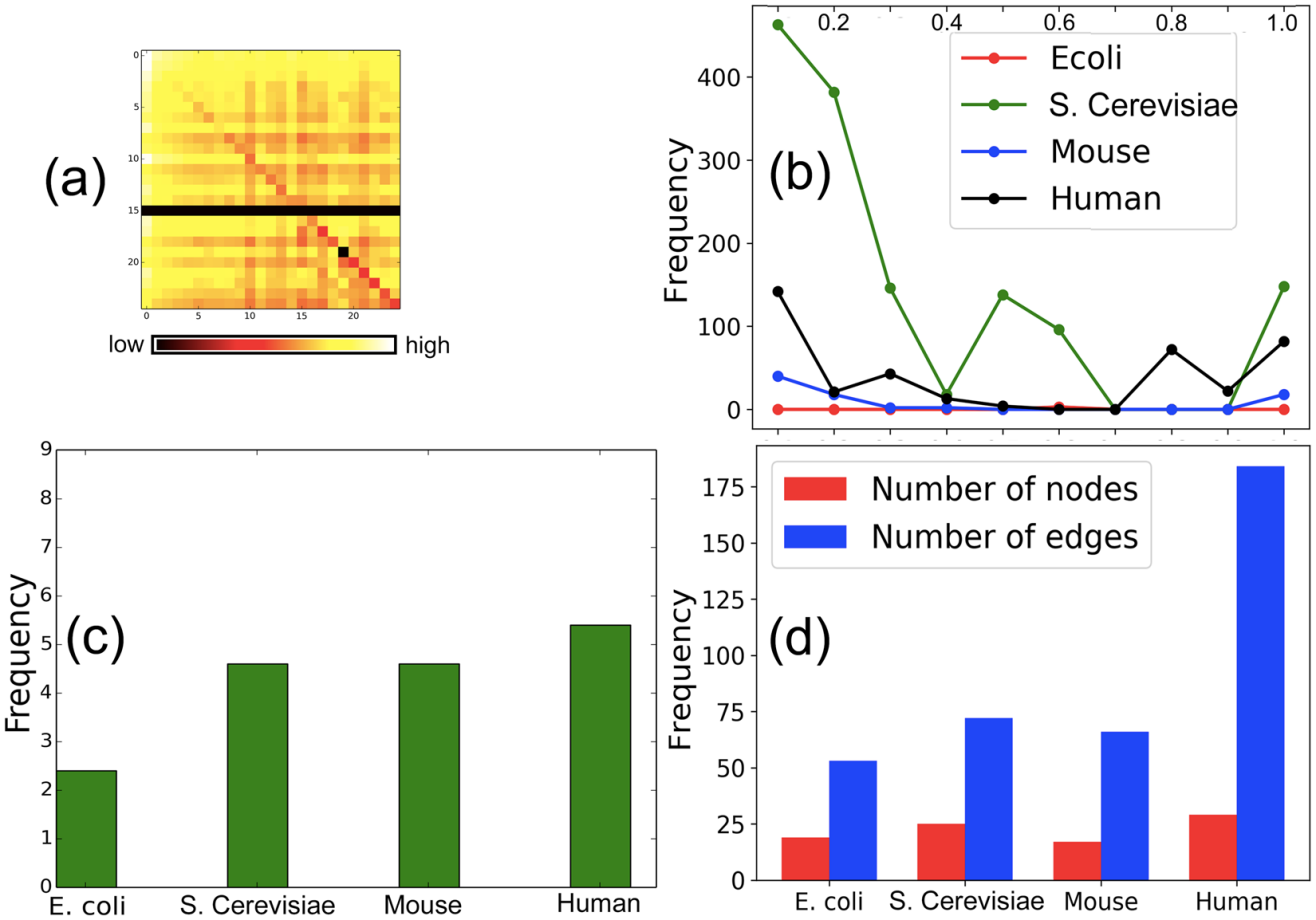
Proximity and cluster formation of the high motif central nodes: (**a**) Average distance between nodes with the FFL motif centrality <25 in human TRN, (**b**) Frequency of the intermediary nodes connecting high FFL motif central nodes, (**c**) Average participation of high motif central nodes in tier 2 in each others’ motif clusters, and (**d**) Large number of FFL motif central nodes are directly connected to one another.

Considering the direction of edges across tiers, we infer that FFL motifs can exist in the forms (a) ${T}_{1}\to {T}_{2}\to {T}_{2}$, ${T}_{1}\to {T}_{2}\to {T}_{3}$, ${T}_{2}\to {T}_{2}\to {T}_{2}$, ${T}_{2}\to {T}_{2}\to {T}_{3}$ (as marked in Fig. 3(a) in white, green, blue and magenta colors). Figure 4(a) depicts that majority of FFL motifs exist among ${T}_{2}\to {T}_{2}\to {T}_{3}$ and ${T}_{2}\to {T}_{2}\to {T}_{2}$. Next, we classify the high NMC nodes across the three tiers to show that the majority of them belong in tier 2 (see Fig. 4(b)). (The implications of these findings are discussed in Sec. 4.1). To study the organization of the high NMC nodes, we first rank the nodes (excluding those with non-zero NMC) in the increasing order of NMC. The role $A$, $B$ and $C$ participation is calculated for two cases: (i) NMC < 100 and (ii) ≥100. In Fig. 4(c,d), we show that, for both the mentioned cases, tier 2 nodes predominantly possess role $A$ and $B$ properties. Second, we show through a heat map (Fig. 5(a)) that the average shortest path length (between node pairs having NMC ≤ 25) tends to decrease as the NMC value increases; this suggests that the motif central nodes (and, by extension, the FFL motifs themselves) lie close to one another. Third, in an attempt to get further insights into the intermediary nodes connecting the high NMC nodes, we generate 10 discrete levels ($0.1,0.2,\ldots ,1.0$) of NMC with respect to the maximum NMC in each TRN topology. For example, if a node belongs to level 1.0, it implies that NMC is between 90–100% of the maximum NMC in a TRN. For each of the $10$ levels, we estimate the frequency of nodes serving as intermediaries in the directed paths connecting the high NMC nodes. Figure 5(b) shows that the low NMC nodes (belonging to level 0.1 and 0.2) predominantly serve as intermediary nodes connecting high NMC nodes belonging in tier 2, although a high number of intermediaries are high NMC nodes (i.e. belong to level 1.0).

#### Clustering among motif central nodes

Given any node in a directed graph, we define its *motif cluster* as the subgraph consisting of the set of nodes and edges participating in FFL motifs containing that node. Gorochowski *et al*. showed that FFL motifs in complex networks exist in clusters, i.e., there exists a great deal of overlap in terms of shared nodes between a pair or set of FFL motifs^20^. For instance, in Fig. 3(b), two FFLs form a cluster by sharing two nodes. Furthermore, we estimate the average participation of $10$ highest NMC nodes of tier $2$ in each others’ motif clusters. Figure 5(c) shows that, on a scale of $0$ to $9$, the average participation of high NMC nodes is around $5$ (which is considered very high in^20^), suggesting that high NMC nodes often share FFL motifs. Finally, we generate subgraphs solely consisting of high NMC nodes and edges connecting them (i.e, through single hop connections). Figure 5(d) shows that particularly in mouse and human TRN, several high NMC nodes are directly connected to one another.

### Communication efficiency and fault-tolerance

This experiment combines results on the effect of role *A* and *B* motif centrality on communication efficiency and fault-tolerance in *E*. *coli*, *S*. *cerevisiae*, human and mouse TRNs. We primarily employ two metrics, namely, network efficiency ($Z$) and infection spread using SIR epidemic model. Specifically, we observe how network efficiency is affected when $0.1 \% ,0.2 \% ,\ldots ,0.5 \%$, role *A* and *B* motif central nodes from the input TRN graph are knocked off. Second, we use the SIR epidemic model (where 0.005% of total TRN nodes are infected) to show the information dissemination potential of role *A* and *B* motif central nodes. We compare the effect of motif central nodes on $Z$ and epidemic spread with those of randomly chosen nodes and nodes with high out-degree (i.e. hubs). The idea is to discern whether the capacity of a node to act as an effective information spreader is a function of its high out-degree or motif centrality. To this end, we perform the following five types of node selections:

- *Role* $A$ *motif central node:* Select nodes based on a likelihood proportional to their role $A$ FFL motif centrality. The probability of selection of node $u$ with role $A$ motif centrality ${\delta }_{A}(u)$ is given by $\frac{{\delta }_{A}(u)}{{\sum }_{v\in V}\,{\delta }_{A}(v)}$.
- *Role* $B$ *motif central node:* Select nodes based on a likelihood proportional to their role $B$ FFL motif centrality. The probability of selection of node $u$ with role $B$ motif centrality ${\delta }_{B}(u)$ is given by $\frac{{\delta }_{B}(u)}{{\sum }_{v\in V}\,{\delta }_{B}(v)}$.
- *Total motif central node:* Select nodes based on a likelihood proportional to their total FFL motif centrality (i.e. sum total of roles $A$, $B$ and $C$). The probability of selection of node $u$ with motif centrality $\delta (u)$ is given by $\frac{\delta (u)}{{\sum }_{v\in V}\,\delta (v)}$.
- *Random node:* Select randomly from the node set of each topology.
- *Hub node with low role* $A$ *motif centrality:* Select nodes with high out-degree and low role $A$ motif centrality, i.e. with likelihood proportional to ratio of node out-degree to role $A$ motif centrality. The probability of selection of node $u$ with role $A$ motif centrality ${\delta }_{A}(u)$ and out-degree ${d}_{O}(u)$ is given by $\frac{{d}_{O}(u)/{\delta }_{A}(u)}{{\sum }_{v\in V}\,{d}_{O}(v)/{\delta }_{A}(v)}$.

**Figure 6:**
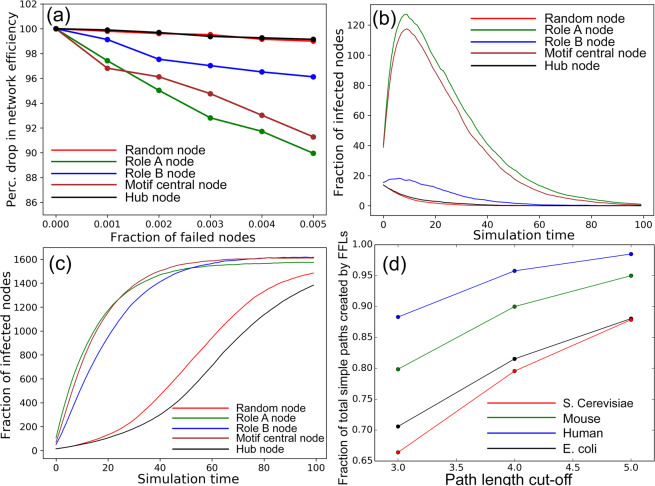
Role of motifs in communication efficiency and fault-tolerance: (**a**) Percentage of network efficiency during node failure in human TRN, Infection propagation using SIR model in human TRN for (**b**) $\frac{\beta }{\gamma } < 1$ ($\beta =0.02,\gamma =0.1$) (**c**) $\frac{\beta }{\gamma } > 1$ ($\beta =0.1,\gamma =0.02$) and (**d**) Fraction of total simple paths created in TRN by the direct and indirect link in FFL motifs.

Figure 6(a) shows that the failure of random and hub nodes of low role $A$ motif centrality in human TRN causes the least dip in network efficiency, followed by role $B$ motif central node and total motif central nodes, whereas knocking off role $A$ motif central nodes leads to the maximum drop in network efficiency implying that these nodes play a significant role in rendering fault-tolerance to TRN topologies. Figure 6(b,c) show the plots for the mean evolution of infected individuals in human TRN for $\frac{\beta }{\gamma } < 1$ and $\frac{\beta }{\gamma } > 1$. In the first SIR epidemic scenario (i.e., $\gamma  > \beta$), the spread of infection peaks and subsequently recedes when all nodes recover; in the second scenario (i.e., $\beta  > \gamma$), the spread of infection increases until it plateaus. Information propagates the fastest for role $A$ motif central nodes, followed by total and role $B$ motif central nodes. Random node failure and hub nodes with low role $A$ centrality have the least spread. Thus, role $A$ motif central nodes emerge as better information spreaders than high out-degree hub nodes with low role $A$ motif centrality. Similar results for communication efficiency and fault-tolerance were observed in mouse TRN and shown in Appendix E and F of the Supplementary Material.

#### Role *A* motif central nodes information spreaders

We apply the following two measures to understand why role $A$ motif central nodes exhibit high spreading potential in Sec. 3.2: (1) *Correlation study with graph centrality*: We evaluate the correlation between role $A$ NMC nodes and the centrality metrics such as *degree*, *closeness* and *betweenness*. For each correlation, we find the scatter plot and apply nonlinear regression to obtain best fit lines. The plots (in Section G of Supplementary Materials) show a moderate to strong correlation between normalized role $A$ NMC and betweenness, degree and closeness centrality, showing that role $A$ NMC nodes are good information spreaders. (2) *Correlation study with k shell decomposition:* As mentioned, Kitsak *et al*. showed that the most efficient information spreaders are located in the inner core of the network (i.e. high k value), fairly independently of their degree^59^. The high correlation between role $A$ NMC and k shell property (shown in Appendix G of Supplementary Material) evidences that role $A$ NMC nodes belong in highly-connected neighborhoods in the core of the TRNs, making them rapid information spreaders.

#### Participation of FFL motifs in simple paths to show their information dissemination potential

We gauge the participation of the two paths between source *S* and target *T* of FFL motifs (refer Fig. 1) in simple paths of varying lengths in TRNs. To this end, we apply our proposed heuristic (Materials and Methods Sec. 2.2.2) with maximum considered path-length $pLimit=3,4,5$ on TRNs of *E*. *coli*, *S*.*cerevisiae*, human and mouse TRN topologies. Our evaluation shows that the path participation varies from $\mathrm{65 \% }$ for path-length cut off 3 to over $\mathrm{95 \% }$ for path-length cut-off 5 (Fig. 6(d)). The high path participation of FFLs (especially for path length cut-off of 5) suggests that the direct and indirect links of FFL motifs create the majority of the *simple paths* (defined in Sec. 2.1), thereby taking part in bulk of the information flow.

### Functional properties of motif central nodes

For each of the four TRN topologies, we first rank the top 10 high NMC nodes in tier 2 that do not feature in the list of top 10 high degree nodes. We then analyze their functional properties in light of their role *A* and *B* centralities. We do not include role *C* because they are more indicative of regulated rather than regulating entities (see Sec. 2.2.1). We take into account another metric, the motif clustering diversity (MCD) (see Sec. 2.2.4), which warrants the role of a node as a global regulator.

#### Motif clustering diversity (MCD) and k shell decomposition

Gorochowski *et al*. argued that although some high NMC nodes within a FFL motif cluster may possess high connectivity, their interactions are often restricted to within the motif cluster, making them unlikely to play a broader role in coordination of many functions across the system. However, since high MCD nodes, by definition, span several motif cluster types, they play a key role in the overall information flow in any network. With regard to k value, nodes possessing high k value are likely candidates for efficient spreaders of information^59^.

We analyze the functional properties of high NMC (and low degree central) tier 2 nodes for human TRN (Table 2); note that similar experiments were also performed for *E*. *coli*, *S*.*cerevisiae* and mouse TRNs and the names and properties of their high NMC tier 2 TFs/genes are included in Appendix H of the Supplementary Materials. For each high NMC node, we report role *A* and *B* centralities, MCD values and the number of signalling pathways (from the KEGG database^60^) it participates in, as an indirect way of highlighting its functional role. *We believe that while role A NMC closely correlates with the information dissemination potential of a node*, *its role in fault tolerance is better quantified by the role B motif centrality*. Finally, we report some findings from published literature on the fault tolerance achieved by some of these high role *B* NMC nodes. Note that the biological experiments do not directly quantify information spread or fault tolerance of TRNs in terms of network level metrics like connectivity or network efficiency; hence, the evidence cited here is only an indirect measure where we focused on master regulators to signify information spread and cell susceptibility or stress response to signify fault tolerance.

**Table 2:** Functional properties of high motif central nodes in human TRN.

TF/Gene	Roles		MCD	KEGG Pathways	k value
	A	B			
ESR1	248	245	12	8	11
HIF1A	130	208	12	11	11
FOS	98	161	12	42	11
HDAC1	182	103	12	14	11
BRCA1	86	144	12	7	11
EGR1	105	163	12	7	11
STAT1	86	108	12	26	11
GATA1	82	94	11	0	9
RB1	88	73	12	89	11
GATA3	67	83	12	5	10

#### Human TRN

Functional properties of high NMC nodes in human TRN demonstrate both role *A* and role *B* properties (Table 2) in similar proportions. Most of these high NMC (and low degree central) nodes exhibit high MCD values and hence act as master regulators. Also note that the high MCD of the high NMC nodes corroborate the clustering tendency of FFLs we demonstrate in Sec. 3.1.2. Since signalling pathways in human TRN are better documented in KEGG, we found better evidence of large signalling pathway involvement for these nodes barring some cases such as GATA1. In the following, we report the involvement of seven of these nodes in fault tolerance from published literature as a means for biological validation. Again, we also document the *k*–shell values of the TFs/genes. We observe that majority of the nodes reported in Table 2 possess the highest k value (equal to $11$), while GATA1 and GATA3 have k values equal to 9 and 10, respectively.

- ESR1 serves as a key TF in regulating CYP3A4, a drug metabolizing enzymes in liver^61^, and its disruption in male rats leads to the change in expression of genes regulating hepatic lipid and carbohydrate metabolism^62^. Moreover, knockout of ESR1 alters the development of stress-related responses as well as psychomotor responses in male and female mice^63^.
- HIFs serve as master regulators of stemness properties and altered metabolism of cancer and metastasis-initiating cells. Activated HIFs lead to the expression of gene products that are responsible for self-renewal, survival, energy metabolism, invasion and metastases of cancer cells, angiogenic switch and treatment resistance^64^. In another work, HIF1 activation has been shown to be related with a variety of tumors and oncogenic pathways^65^, while its deletion increases susceptibility of beta cells to viral infections and toxins, thereby enhancing the occurrence of autoimmune diabetes in mice^66^.
- c-Fos regulates cell proliferation and differentiation and its deregulation has been shown to cause oncogenic progression. Moreover, 40% of c-fos −/− mouse embryos survive until birth, which exhibit an average lifetime of 6–7 months, growth retardation, severe osteopetrosis, delayed or absent gametogenesis and altered haematopoiesis^67^.
- HDAC inhibitors play a part in proliferation, apoptosis and inflammation. Specifically, HDAC1 and HDAC2 regulate intestinal inflammatory response in mice through intestinal epithelial cell proliferation and differentiation^68^; they are also master regulators in epidermal development as they cause homogeneous expression of both proteins in epidermal nuclei^69^. There is reduced expression of HDACs in stress-susceptible mice leading to deficiency in cognitive capabilities^70^. Studies report high HDAC1 levels in blood samples from human patients experiencing early life stress and schizophrenia^71^.
- BRCA1 is a master regulator of heart function and its loss in mice causes deterioration in cardiac remodelling, ventricular function and higher mortality in response to stress^72^. Additionally, it is associated with the transcription of several genes in the anti-oxidant response pathway resulting in regulation of oxidative stress^73^. BRCA1 maintains genomic stability by regulating gene expression, chromatin remodeling, DNA repair, etc^74^. Consequently, BRCA1-deficient embryos are reported to be more vulnerable to ethanol-initiated DNA damage and embryopathies^75^.
- EGR1 is a master regulator in several biological processes, regulating tumor suppressor genes and inhibiting growth of several human cancer types^76^. EGR1 is also a stress response gene, that affects inflammation and tissue repair. Knockout experiments on mice show that hepatocyte EGR1 helps maintain hepatic insulin response, and as a result, the loss of EGR1 in hepatocytes can contribute to liver steatosis leading to non-alcoholic fatty liver disease development^77^.
- STAT1 has been shown to be a master regulator of Pancreatic *β*-Cell Apoptosis and Islet Inflammation; it regulates gene networks linked with cell cycle, signal transduction, apoptosis, endoplasmic reticulum stress, and inflammation in *β*-cells^78^. Also, STAT1−/− mice showed a marked increase in PGC1 *α* – a master regulator of mitochondrial biogenesis^79^. In another experiment, STAT1 knockout mice exhibited high vulnerability pulmonary mycobacterial infection^80^. In studies pertaining to role of STATs in stress response, STAT1 and STAT5 showed complementary effects; while STAT1 activation by hypoxia-reperfusion injury activates cell death pathways, STAT5 activation leads to cell survival pathways^81^.
- GATA1 is a master regulator of hematopoiesis responsible for transcription of genes encoding the essential autophagy component microtubule-associated protein^82,83^. Studies on GATA1 knockout mice show GATA1 to be the key regulator for erythropoiesis, regulating gene expression associated with erythroleukemia cells and normal erythroid progenitors^84,85^.
- RB is a master regulator of the cell cycle, which plays the role of tumor suppressor with important chromatin regulatory functions that affect genomic stability^86^. Rb knockout mice were lacking in the organization of osteoblast layers^87^. Studies on RB w.r.t. oxidative stress showed that RB is responsible for the regulation of stroll microenvironment^88^.
- GATA3 is a master regulator and a well-studied drug target for ovarian carcinoma^89^. Conditional knockout studies show GATA-3 to be crucial for optimal T- helper type 2 (Th2) cytokine production that contribute towards the mediation of allergic and asthmatic disease^90^, while GATA3 also takes part in mediation of survival signals in osteoblasts^91^.

We report a literature review on the role of the 10 high NMC tier 2 nodes in global regulation, stress response and the effect of their knockout on the normal function of an organism. Although we report the 10 TFs/genes in the context of human TRNs, in most cases their roles have only been confirmed in mouse in the published literature. Clearly their functional properties in humans should be investigated in the future. TFs such as ESR1, HDAC, BRCA1, EGR1, STAT1 and RB play dual roles of master regulation as well as stress response which may indirectly explain their high role *A* and *B* values in Table 2. Recall from the discussion in Sec. 2.2.1, roles *A*, *B* and *C* centralities are distinct, i.e., the same node cannot play both roles *A* and *B* in the same FFL. Clearly, the TFs playing the combined role of master regulation and stress response are controlling gene expression through different pathways in the TRN. These observations raise the interesting question on whether the analysis of FFL motif centrality can lend epigenetic insights leading to the identification of potential drug targets for specific disease conditions.

## Discussions

The novelty of this manuscript in terms of method firstly lies in pinpointing the organization or connectivity among individual FFL motifs in TRNs in light of a node level measure called FFL motif centrality and its different roles (i.e., role *A*, *B* and *C*). The other notable contribution with regard to method is the elucidation of the topological and functional properties of role *A* and *B* motif central nodes in TRN by means of standard network science based metrics (such as network efficiency, path enumeration, epidemic spread, motif clustering diversity, k shell decomposition, etc.) and biological metrics (biological pathways, master regulation regulation and stress response). In the remaining manuscript, we propose a hub-and-spoke architecture to reconstruct the TRNs solely with tier 2 FFL motif central nodes. We intuit that this proposed architecture can be generalized to analyze the information spread and fault-tolerance of any complex network topology; then we present the future biological implications of high NMC TRN nodes in the context of identification of biomarkers and drug targets for specific disease conditions.

### Network implication

We re-conceptualize the TRN topology in terms of the motif centrality of nodes. Specifically, we analyze the implications of the organization of high NMC nodes on the information dissemination in TRN. To this end, we construct a new architecture, wherein we focus on the high NMC nodes in tier 2 in the three tier TRN topology (discussed in Sec. 3.1 and depicted in 7(a)). The reason behind investigating tier 2 is that it contains the highest NMC nodes (as shown in Fig. 4(b) as well as in Table 3).

**Table 3:** Average node motif centrality of TRN nodes in each tier.

TRN type	E. coli	S. cerevisiae	Human	Mouse
Tier 1	$2.3$	$15.6$	$0.5$	$0.3$
Tier 2	$27.7$	$63.3$	$30.0$	$13.6$
Tier 3	$1.2$	$0.9$	$2.2$	$1.0$

**Figure 7:**
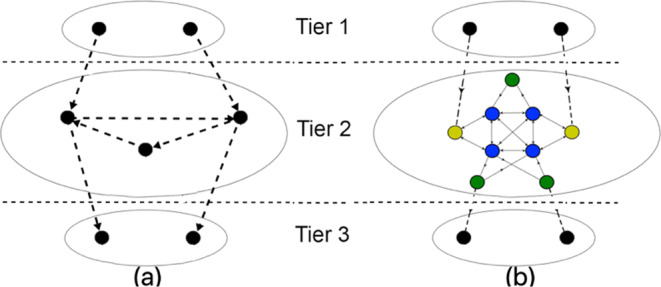
Organization of TRN nodes: (**a**) three tier topological characterization, and (**b**) Hub-and-spoke TRN architecture.

Figure 7(b) shows the schematic of the proposed *hub*-*and*-*spoke TRN architecture*, where there exists a few high NMC nodes (colored blue) that form cliques among themselves, while the other type of high NMC nodes (colored green) are connected to some (but not all) blue nodes. We infer that both the green and blue nodes are connected through bidirectional edges leading to full duplex data flow. There exists a third node type (colored yellow) that serve as intermediaries between blue nodes. Thus, the *spokes* consist of the nodes in tiers 1 and 3 lying at the periphery of the network, while the high NMC tier 2 nodes form a hierarchical hub architecture that receive information from the tier 1 and forward them to tier 3. The high k shell values of the high NMC in tier 2 (reported in Tables 1 and 2) yield further evidence that such nodes form the TRN core. Note that the new architecture differs from a typical hub-and-spoke architecture, where the hubs are the high degree nodes; however, the high NMC tier 2 nodes, forming the hub, are not necessarily the degree central nodes. For ease of understanding, we term such high NMC tier 2 nodes *motif hubs*. (Refer to the actual layout of tier 2 in human TRN in Appendix I of the Supplementary Material).

Just like in any hub-and spoke-architecture, the bulk of the TRN nodes (residing in tiers 1 and 3) communicate with the motif hubs. This minimizes the number of links, making the TRNs extremely sparse (i.e. having extremely low graph density *D*) as shown in Table 1. The motif hubs colored blue demonstrate particularly high role $A$ property, acting as the information spreaders in the network. The green nodes provide fault tolerance against the failure of the blue nodes. The green nodes typically have lower degree than the blue nodes and provide a cost versus information spread trade-off, playing a crucial role only when the network is under stress. Finally, the yellow nodes provide edge level fault tolerance; these nodes generally exhibit a high role *B* property and activate the indirect paths of the FFL when the direct paths are congested or error prone.

From an engineered network perspective, such a hierarchical hub-and-spoke design motivates several directions in constructing efficient networking protocols. For any efficient dynamic routing protocols, let us consider two networking scenarios. Under normal conditions, the next hop neighbor can be chosen as a function of high residual energy, shortest path length to the destination or high role *A* property. However, under stress, a combination of residual energy, shortest path length, role *A* and role *B* properties can collectively be used to determine the next hop neighbor to guard against both node and edge failures.

### Biological implication

In Sec. 3.3, we show the nodes having high role A and B motif centrality, such as HIFs, HDAC, BRCA1, EGR1, STAT1, GATA1 and GATA3, and also report their functions as master regulators in liver function, cell proliferation, tumor suppression and genomic stability, etc. as well as stress response. At present, a popular tool to ascertain the function of any gene is to perform knockout experiments which causes phenotypic variability in cells through inhibition of specific genes^92^. Knockout experiments, showing the role of P450 in offsetting carcinogenic effects of chemicals^93^ or that of TAp73 knockout in genomic stability^94^, suggest that knockouts may be employed to identify genes regulating important cell functions and help with cell stability before developing therapeutics to target the specific gene products^95^. However, it has been shown that effectiveness of knockout targets are challenged by a variety of factors such as genetic backgrounds and culturing conditions^96^. For instance, the purinergic P2X7 receptor expressed by bone cells has been reported to help in bone formation, however the bone phenotype of the P2X7-/- mice is greatly influenced by their genetic background^97^. Keeping in mind the complexities in knockout experiments, we hypothesize that the connection between the FFL centrality of a TF/gene and its topological and functional role in TRNs warrants further investigation and can be used to identify biomarkers and drug targets for specific diseases^78,80,98–101^.

## Supplementary information

Supplementary Material.
