# Supplementary material for: Motifs enable communication efficiency and fault-tolerance in transcriptional networks
Source: Sci Rep. 2020 Jun 15;10:9628. doi: 10.1038/s41598-020-66573-x (PMC7296022; doi:10.1038/s41598-020-66573-x)
Supplement: Supplementary file 1 — Supplementary Material. [file 41598_2020_66573_MOESM1_ESM.pdf]

Motifs enable communication efficiency and fault-tolerance in transcriptional networks

Satyaki Roy, Preetam Ghosh, Dipak Barua, Sajal K. Das

March 18, 2020

1 Appendix A

Table 1 summarizes the abundance of feed forward loops (FFLs) and feedback loops (FBLs) motifs, showing that FFLs outnumber FBLs in all the four TRNs, namely, *E. coli*, *S.cerevisiae*, human and mouse.

Table 1: Abundance of FFLs and FBLs motifs in TRN

TRN type	FFL	FBL
E. coli	4798	12
S. Cerevisiae	4115	39
Human	7557	789
Mouse	4328	492

2 Appendix B

FFLs participate as subgraphs in larger TRN motifs. In Fig. 1, as shown in case of *E. coli* TRN, FFL motifs are contained within 4, 5 and 6 node motifs.

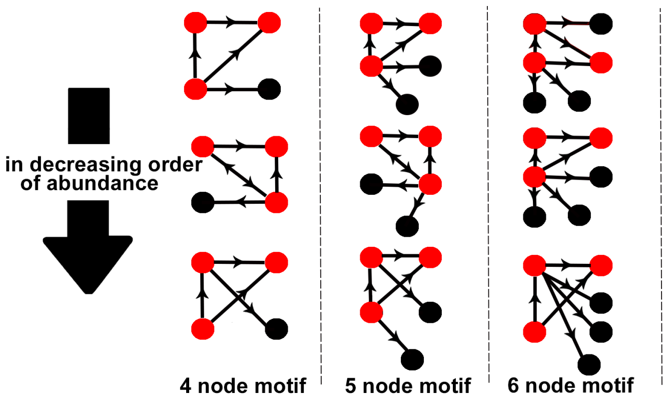

Figure 1: 4,5 and 6 node motifs in *E. coli* TRN contain FFL motifs

3 Appendix C

In this section, we propose a simple heuristic to analyze to what extent the direct and indirect links of FFL motifs contribute towards creating a simple paths in TRNs. Let us consider a path $p = \{u_1, u_2, \cdots, u_n\}$. The path may contain several FFL motifs with edges $e(u_i, u_{i+1})$ as direct links. Recall that $\phi_d(e(u_i, u_{i+1})$ is the number of nodes v such that $e(u_i, u_{i+1})$ is a direct link in the FFL motif (u_i, v, u_{i+1}).

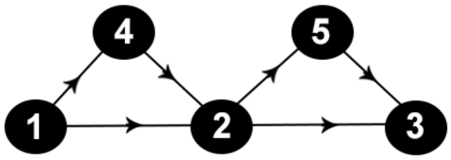

Figure 2: Path p consisting of several FFL motifs

In Fig. 2, we have $p = \{1, 2, 3\}$, where edges $e(1, 2)$ and $e(2, 3)$ are direct links to FFL motifs $(1, 4, 2)$, $(2, 5, 3)$. Thus, $\phi_d(e(1, 2)) = \{4\}$ and $\phi_d(e(2, 3)) = \{5\}$. We next apply an enumerative strategy to determine the number of paths created by FFL motifs: given a path p, replace each direct link (u_i, u_{i+1}) by an indirect path $\{u_i, v, u_{i+1}\}$ to get a new path p' created by FFL motifs (Algorithm 1).

for $e(u_i, u_{i+1})$ **do**
  $p' = \{u_1, u_2, \cdots, u_i, v, u_{i+1}, \cdots, u_n\}\ \forall v \in \phi_d(e(u_i, u_{i+1}))$;
end

Algorithm 1: Enumerative approach

Using this enumerative approach, for $p =< 1, 2, 3 >$, we obtain new paths $p' = \{1, 4, 2, 3\}, \{1, 2, 5, 3\}, \{1, 4, 2, 5, 3\}$. In a path $p = \{u_1, u_2, \cdots, u_n\}$, number of simple paths between u_1 and u_n formed due to FFL motifs is given by:

$$\pi = \prod_{i=1}^{n} (|\phi_d(e(u_i, u_{i+1}))| + 1) \tag{1}$$

Going back to Fig. 2, since $|\phi_d(1, 2)| = 1$ and $|\phi_d(2, 3)| = 1$, number of simple paths π between 1 and 3 $= (|\phi_d(1, 2)| + 1) \times (|\phi_d(2, 3)| + 1) = 4$.

Redundancy in path enumeration: Consider another subgraph G (Fig. 3), having three paths between nodes 1 and 2, i.e., $p_1 = \{1, 3, 2\}$, $p_2 = \{1, 4, 2\}$ and $p_3 = \{1, 3, 4, 2\}$., yet our enumeration equation (Eq. 1) returns 4 paths. This is because path $p_3 = \{1, 3, 4, 2\}$ is common to both direct links $e(1, 4)$ and $e(3, 2)$ and is counted twice. Evidently, Eq. 1 may have redundancy in path enumeration if two motifs share an indirect link. Based on these observations, *we propose a simple heuristic to determine the fraction of simple paths created due to the direct and indirect path of FFL motifs.* Details of the algorithm (Algorithm 2) are discussed below. We present an illustration of the algorithm for subgraph G (shown in Fig. 3).

Illustrative example: Here for each pair of nodes $u, v \in V$, we consider a **score** as the ratio between the number of paths produced as a result of the direct and indirect path of FFL motifs to the total number of paths.

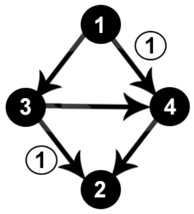

Figure 3: Example subgraph G with edge ϕ_d values shown in circles

Paths between node pair $(1,2)$*:* There are 3 paths namely, $1 \rightarrow 3 \rightarrow 2$, $1 \rightarrow 4 \rightarrow 2$ and $1 \rightarrow 3 \rightarrow 4 \rightarrow 2$ all of which are generated as a result of direct and indirect path of FFLs $(1,3,4)$ and $(3,4,2)$. **Score:** $\frac{3}{3}$.

Data: Graph G, *pLimit*
Result: $score = \frac{C}{\pi}$
// Calculate $\phi(e)$ for all $e \in E(G)$;
// Total paths count ;
$\pi = 0$;
// Total paths count created by FFLs ;
$C = 0$;
for $u \in V(G)$ **do**
  for $v \in V(G)$ **do**
    P : List of simple paths between u and v of length $\leq pLimit$;
    $\pi = \pi + |P|$;
    for $p \in P$ **do**
      Let $p =< u, u_2, u_3, \cdots, v >$;
      P' : All possible paths between u and v generated using ϕ values using enumerate approach of length $\leq pLimit$;
      $C = C + |P'|$;
      $P = P - P'$;
    end
  end
end

Algorithm 2: Path enumeration

Paths between nodes $(1,3)$ *and* $(1,4)$*:* There is 1 path namely, $1 \rightarrow 3$ which is not using the direct and indirect path of FFL $(1,3,4)$. Similarly, there are two paths between $1 \rightarrow 4$: $1 \rightarrow 4$, $1 \rightarrow 3 \rightarrow 4$ that use the FFL $(1,3,4)$. **Scores:** $\frac{0}{1}$ and $\frac{2}{2}$.
Paths from node 2*:* Node 2 does not have any outgoing edges. Scores for node pairs $(2,1)$, $(2,3)$ and $(2,4)$ are all $\frac{0}{0}$.
Similarly, scores for paths between node pairs $(3,1)$, $(3,2)$, $(3,4)$ are $\frac{0}{0}$, $\frac{2}{2}$, $\frac{0}{1}$; and for $(4,1)$, $(4,2)$ and $(4,3)$ are $\frac{0}{0}$, $\frac{0}{1}$ and $\frac{0}{0}$.
Total score, $\frac{3+0+2+2+0+0}{3+1+2+2+1+1} = 0.7$, implies that 70% of the total paths between all pairs of nodes utilize the direct and indirect paths of FFL motifs $(1,3,4)$ and $(3,4,2)$. Here we propose a simple heuristic (Algorithm 2) to determine the ratio between total number of simple paths created by FFL motifs to the total number of paths between all pair of nodes, while discounting the redundancy in path enumeration.

Algorithm description: It takes two input parameters: directed input graph G and maximum considered path-length *pLimit*. For every pair of nodes u and v such

that v is reachable from u, it uses the Python Networkx [1] to determine the list of simple paths P of length $\leq pLimit$. For every path p in list P, the algorithm applies the enumerative approach to list all simple paths P' created from path p via FFL motifs. The heuristic handles redundancy in path enumeration by removing all paths in P' from P. Finally, the algorithm returns the **score** as the ratio between total paths created by FFL motifs, C, and total number of simple paths (of length less than or equal to $pLimit$), π.

4 Appendix D

Here we briefly discuss how we calculate the metrics used to validate the functional properties of motif central nodes in TRN.

4.1 Motif clustering diversity

For any node, one can extract all FFL motifs that contain the selected node as a member. For each pair of FFL motifs that share at least one node, it is possible to create 12 possible configurations (shown in Fig. 4). *Motif clustering diversity (MCD) is defined as the number of different motif clustering types (i.e., configurations) that a node takes part in.* Its value ranges between 0 and 12.

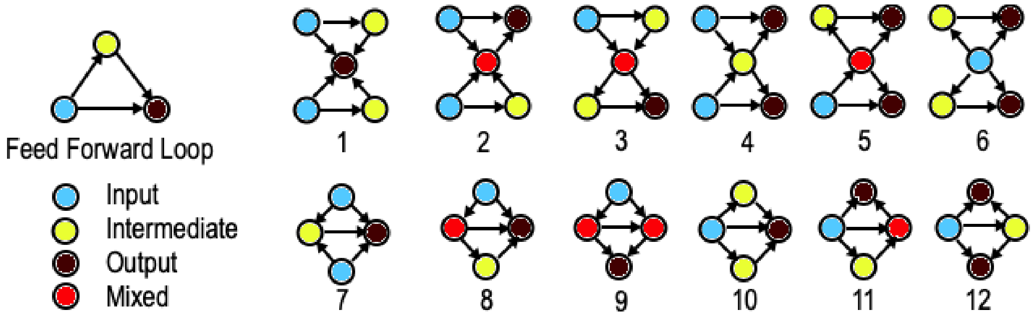

Figure 4: 12 types in Motif Clustering Diversity (MCD) (taken from [2])

4.2 Biological pathways

A biological pathway is a series of actions among molecules in a cell that enable interaction among the genes, molecules and cells. Such a pathway can trigger the assembly of new molecules, such as a fat or protein [3, 4]. Pathways can also turn genes on and off, or make a cell move. There are different types of biological pathways that can help in signal transduction, gene regulation and metabolism.

4.3 k shell decomposition

k shell decomposition is the process of pruning of nodes with the lowest degree in an undirected graph. Each step of pruning is denoted by the variable $k = 1, 2, 3 \cdots$. After every step of pruning, the resultant *k-core subgraph* is the maximal subgraph such that every vertex has degree at least k. The integer value k is attached to the set of vertices that are part of the k^{th}core but not part of the $(k+1)^{th}$ core. From

the process of calculating k value, it is clear that small k values correspond to nodes in the network periphery and innermost network core corresponds to large k values.

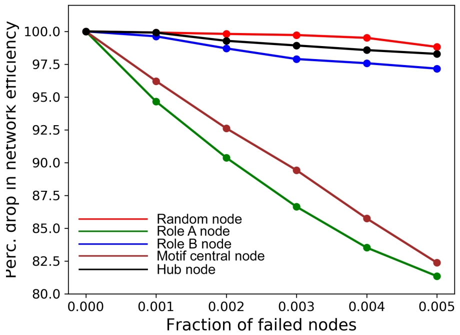

Figure 5: Percentage network efficiency preserved during node failure for *E. coli*

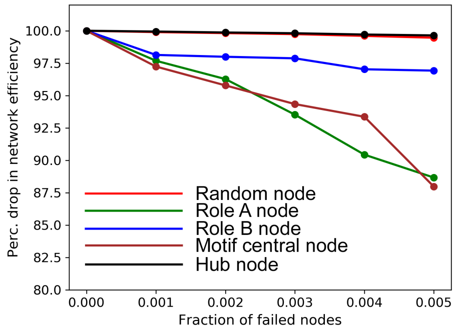

Figure 6: Percentage network efficiency preserved during node failure for *S. Cerevisiae*

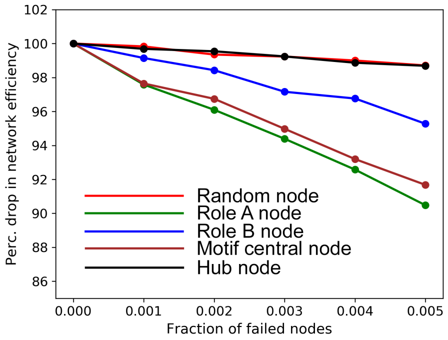

Figure 7: Percentage network efficiency preserved during node failure for mouse

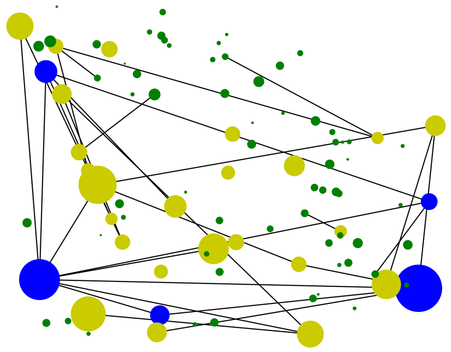

Figure 8: Actual layout of tier 2 nodes in Human TRN

5 Appendix E

As a measure of fault-tolerance (defined in Sec. 2.2.3 of the paper), we study the drop in network efficiency of *E. coli*, *S. Cerevisiae* and mouse TRNs for failure of $0.1\%, 0.2\%, \cdots, 0.5\%$, nodes, respectively. We consider 5 node removal strategies:

- *Random node:* Select randomly from each TRN node set.
- *Role A motif central node:* Select nodes based on a likelihood proportional to their role A FFL motif centrality. The probability of selection of node u with role A motif centrality $\delta_A(u)$ is given by $\frac{\delta_A(u)}{\sum_{v \in V(G)} \delta_A(v)}$.
- *Role B motif central node:* Select nodes based on a likelihood proportional to their role B FFL motif centrality. The probability of selection of node u with role B motif centrality $\delta_B(u)$ is given by $\frac{\delta_B(u)}{\sum_{v \in V(G)} \delta_B(v)}$.
- *Total motif central node:* Select nodes based on a likelihood proportional to their total FFL motif centrality (i.e. sum total of roles A, B and C). The probability of selection of node u with motif centrality $\delta(u)$ is given by $\frac{\delta(u)}{\sum_{v \in V(G)} \delta(v)}$.

- *Hub node with low role A motif centrality:* Select nodes with high out-degree and low role A motif centrality, i.e. with likelihood proportional to ratio of node out-degree to role A motif centrality. Thus, the probability of selection of node u with role A motif centrality $\delta_A(u)$ and out-degree $d_O(u)$ is given by $\frac{d_O(u)/\delta_A(u)}{\sum_{v \in V(G)} d_O(v)/\delta_A(v)}$.

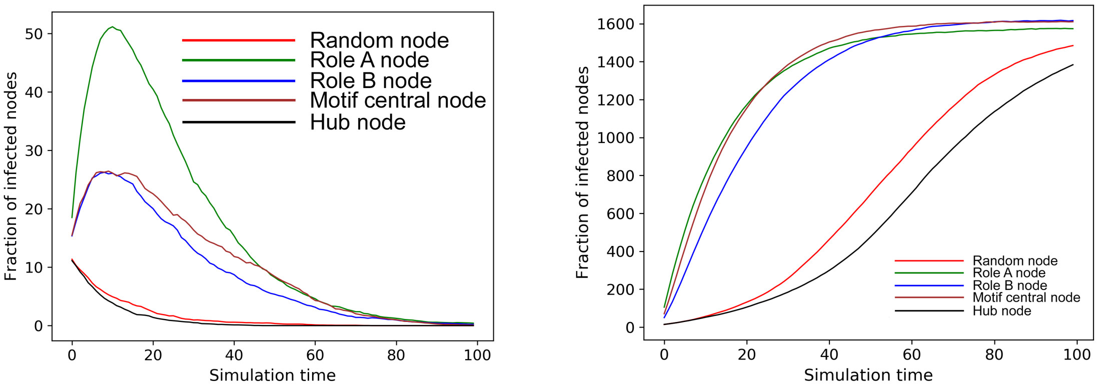

(a) $\beta = 0.02, \gamma = 0.1$ (b) $\beta = 0.1, \gamma = 0.02$

Figure 9: Infection propagation in Mouse TRN

Figs. 5,6 and 7 show that knocking off role A node motif central (NMC) nodes causes the maximum drop in network efficiency in TRNs, explaining the role of A NMC nodes in information spread and fault-tolerance.

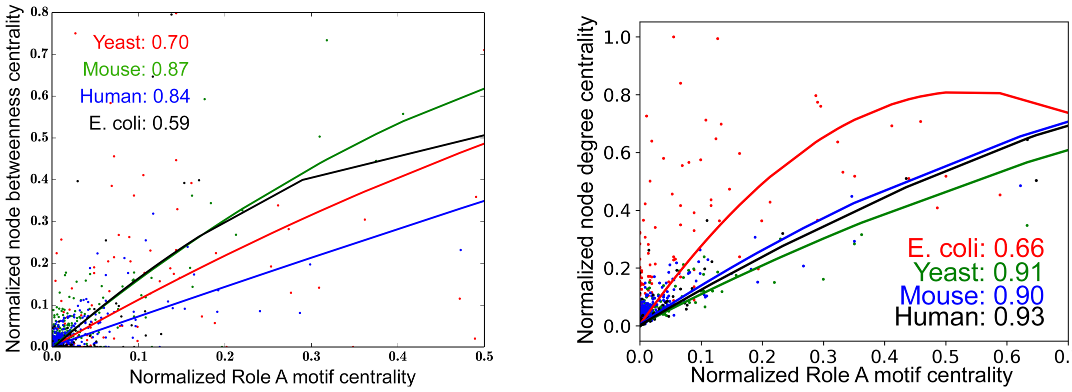

(a) (b)

Figure 10: Correlation between normalized role A motif centrality and (a) betweenness and (b) out-degree centrality. The numbers represent the respective correlation coefficient.

6 Appendix F

Fig. 9 shows the plots for the mean evolution of infected individuals in mouse TRNs. Information propagates the fastest for role A NMC nodes, followed by total and role B NMC nodes, for both (a) $\frac{\beta}{\gamma} < 1$ and (b) $\frac{\beta}{\gamma} > 1$. Random node failure and hub

nodes with low role A centrality exhibit the least spread. Thus, role A NMC nodes emerge as better information spreaders than hub nodes with low role A NMC in a TRN particularly when $\frac{\beta}{\gamma} < 1$, showing that the role A NMC nodes tend to retain the infection for longer duration.

7 Appendix G

We study the correlation between role A NMC and other network centrality metrics. Fig. 10 and 11(a) show that there is a moderate to strong correlation between normalized role A NMC and normalized betweenness, degree and closeness centrality values, explaining why role A NMC nodes are good information spreaders in a TRN.

k shell decomposition: Kitsak et al. showed that the most efficient spreaders are located in the inner core of the network (i.e. high $k-$value), fairly independently of their degree [5]. We know that $k-$core of a graph is the maximal subgraph such that every vertex has degree at least k. Unlike hub nodes that guarantee high number of neighbors, $k-$ shell of a node shows how central a node is with respect to its neighbors. Therefore, a higher $k-$shell value suggests that the node belongs to a more connected neighborhood in the network [6]. Thus, nodes with high $k-$shell value are likely to spread the data more rapidly.

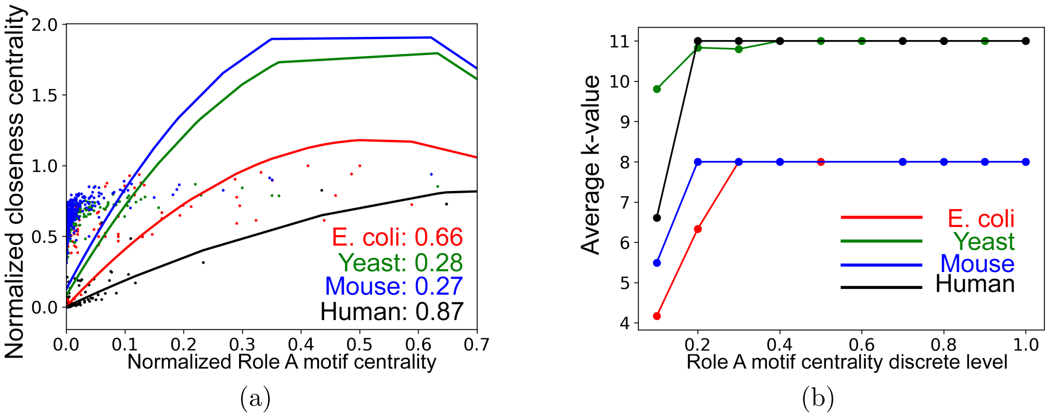

Figure 11: Correlation between normalized role A motif centrality and (a) closeness centrality and (b) average k value.

We discretize the role A NMC into 10 levels $(0.1, 0.2, \cdots .1.0)$. For instance, a value of 1.0 implies that the node role A NMC lies between $90-100\%$ of the maximum role A NMC. Fig. 11(b) shows that the average $k-$ shell value increases with normalized role A NMC and plateaus at 0.2.

8 Appendix H

Here we report the top 10 TFs and genes with high role A and B properties (but not featuring in top 10 high degree nodes) in Escherichia coli (*E. coli*), *S. Cerevisiae* and Mus musculus (mouse) TRN that play a role in fault tolerance. Additionally, we also report the MCD and k values of the respective TFs. It is noteworthy that

most of the chosen nodes exhibit high MCD and k values showing their properties as global regulators and information spreaders in the networks. Also, the highest k value for *E. coli*, *S. Cerevisiae* TRNs and mouse are 8 and 11, respectively.

8.1 Mouse TRN

The functional properties of high NMC nodes in the Mouse TRN are reported in Table 4. Each of the top nodes exhibit appreciable role A and role B type NMC. Although the signalling pathway count for some of these nodes are quite low, they all demonstrate high MCD values to underline their role as global regulators and information spreaders in the network. This could be due to the fact that the signalling pathway participation of all these nodes have not yet been confirmed experimentally in KEGG. Of particular note is the Crebbp gene having slightly lower MCD (of 8), but it actually does participate in an appreciable number of signalling pathways. Since each of these nodes demonstrate appreciable role B centralities, we showcase their involvement in fault tolerance related pathways from published literature in the following; only five genes/TFs are reported in this validation study, while the fault tolerance of the other reported nodes have not been confirmed yet in the literature. With regard to the $k-$shell value, all the nodes discussed in Table 4 exhibit the highest $k-$shell value observed in mouse TRN (equal to 8), suggesting that they are located in the network core and play a role in information spread.

Table 2: Functional properties of high motif central nodes in Mouse TRN.

	Roles				
**TF/Gene**	**A**	**B**	**MCD**	**Pathway**	**k value**
Myc	70	139	12	54	8
Pou5f1	50	82	12	1	8
Nfe2l2	85	76	11	4	8
Crebbp	90	49	8	26	8
Sox2	51	62	11	2	8
Snai1	49	61	11	1	8
Ctnnb1	52	46	12	26	8
Myod1	38	45	12	0	8
Sp7	20	47	11	21	8
Cebpb	43	39	12	4	8

- The MYC proto-oncogene is a gene product that coordinates the transcriptional regulation of a multitude of genes essential to cellular programs required for normal as well as neoplastic cellular growth and proliferation, including cell cycle, self-renewal, survival, cell growth, metabolism, protein and ribosomal biogenesis, and differentiation [7], explaining both role A and role B properties.

- The POU5F1 gene encodes a key regulator that helps maintain pluripotency in embryonic stem (ES) cells [8], and its conditional knockout in mouse primordial germ cells causes apoptosis [9].

- NFE2L2 is considered as a master regulator of cellular homeostasis, and it controls the expression of numerous cytoprotective genes [10]. Additionally, Nfe2l2 knockout mice develop degeneration in the retinal pigment epithelium, suggesting that its deficiency may induce retinal disease [11].

- CREBBP is involved in several cellular processes and functions as a transcriptional cofactor and histone acetyltransferase (HAT), and CREBBP mutations are found in many human cancers [12].

- SOX2 is a master regulator of both pluripotent embryonic stem cells (ESCs) and multipotent neural progenitor cells (NPCs) [13]. SOX2 deficient mice show poor behavioral adaptation to environment [14].

- Snai1 expression has been linked to enhanced cellular survival in mouse embryos and cell lines [15]; in chemoresistance in tumors, Snai1 expression can enhance resistance of cells to stress-induced apoptosis [16]. Snai1 expression in keratinocytes can also enhance survival following stress [17, 18] thereby demonstrating its potential role B properties.

- Knockout of CTNNB1 in human cell lines lead to an inhibition in adhesion and cell proliferation [19].

- MyoD is a master regulator of skeletal myogenesis due to its ability to initiate the myogenic program in several cells [20]. It was also reported to enhance glutathione production and protect against oxidative stress by positively regulating many antioxidant genes [21].

- SP7 is a master regulator for a variety of genes during differentiation of osteoblasts [22]. The deletion of Sp7 in postnatal mice disrupts osteoblast differentiation and severely impairs bone formation [23].

8.2 *E. coli* TRN

- Fur, along with BasR and CysB, coordinates serum metabolic response in *E. coli* [24].

- GadEWX (i.e., GadE, GadW and GadX) regulate glutamate-dependent acid resistance (GDAR) system in *E. coli* [25]. They are also responsible for acid resistance, stress response, etc.

- GadE is a key regulator for GadA and GadB, which can contribute to the ability of *E. coli* to withstand acid stress [26].

- MarA was reported to play a role in adaptive response. marA is one of the global regulators responsible for several regulatory mechanisms necessary for the adaptive response. MarA responds to stimuli such as changing pH, presence of antibiotics, oxidative stressors and organic solvents, etc. [27].

- The Cpx contributes to adaptation to stress that cause envelope protein misfolding [28].

Table 3: Functional properties of high motif central nodes in *E. coli* TRN.

**TF/Gene**	Roles **A**	**B**	**MCD**	**Pathway**	**k value**
Fur	88	133	8	26	6
GadX	88	133	12	0	6
GadE	63	91	11	0	6
GadW	39	50	7	0	6
MarA	50	77	9	19	6
CpxR	71	56	2	2	5
SoxS	61	36	6	0	6
FhlA	42	93	4	0	5
GlnG	45	52	5	1	4
LexA	55	55	0	0	2

- Sox is believed to be associated with some disease conditions in humans [29]. SoxS protein directly regulates oxidative stress genes of the *soxRS* regulon [30].
- LexA is a master regulator contributing to DNA repair [31].

Table 4: Functional properties of high motif central nodes in *S. Cerevisiae* TRN.

**TF/Gene**	Roles **A**	**B**	**MCD**	**Pathway**	**k value**
Yap6	362	369	12	0	11
Rox1	213	193	12	0	11
PHD1	176	188	12	0	11
Swi4	120	242	9	3	11
Nrg1	315	46	7	0	11
Msn4	59	203	12	4	11
Msn2	159	68	9	0	11
Xbp1	20	173	12	0	11
Yap1	72	125	12	0	11
Gcn4	117	74	12	0	11

8.3 *S. Cerevisiae* TRN

- Yap6, along with three other TFs, has been experimentally confirmed to be associated with stress response gene regulation [32].
- Rox1 is associated with adaptive response to hyperosmotic stress [33].
- PHD1 has been reported to be a stress response gene [32]. Experiments to investigate the role of PHD1 in homeostasis in mice suggests PHD1−/− mice

have higher white adipose tissue (WAT) mass (in spite of lower body weight), and poor insulin sensitivity and glucose tolerance [34].

- Nrg1 is a master regulator of myelination [35].
- Msn2/4 is a master regulator in S. Cerevisiae that has been shown to protect the cells from starvation and facilitate regrowth [36].
- Xbp1 contributes to cell proliferation, apoptosis, metastasis, and drug resistance and promotes cell survival. Thus, it is considered to be a biomarker for cancer development and progression. Furthermore, knockout experiments on mice suggest that Xbp1 helps restore homeostasis in the liver in response to endoplasmic reticulum (ER) stress [37].
- Yap1 has been reported to regulate cell survival and tissue morphogenesis [38] and control the oxidative stress response regulon of at least 32 proteins [39].
- Gcn4 is a master regulator under conditions of amino acid starvation and environmental stresses. Specifically, it also participates in the response to peroxide stress in *S. Cerevisiae* [40].

9 Appendix I

Fig. 8 shows the actual layout of the tier 2 nodes in human TRN, where the bidirectional edges between nodes are shown as undirected links and the unidirectional edges (existing mostly between blue and green nodes) are not shown in the interest of clarity. There is one point of difference: instead of a single hub (in hub-and-spoke architecture), there is a set of blue, yellow and green hubs comprising of high NMC nodes, called *motif hubs*. The yellow nodes provide redundancy, and the green nodes enable information flow between the high NMC nodes. Such a hub-and-spoke architecture has also been reported independently in the brain network.

References

[1] Hagberg, A., Swart, P. & S Chult, D. Exploring network structure, dynamics, and function using NetworkX. *Los Alamos National Lab.(LANL), Los Alamos, NM (United States)*. (2008)

[2] Gorochowski, T., Grierson, C. & Dibernardo, M. Organization of feed-forward loop motifs reveals architectural principles in natural and engineered networks. *Science Advances*. **4(3)**, eaap9751 (2018)

[3] National Human Genome Research Institute. *https://www.genome.gov/about-genomics/fact-sheets/Biological-Pathways-Fact-Sheet*.

[4] KEGG PATHWAY Database. *http://www.genome.jp/kegg/pathway.html*.

[5] Kitsak, M., Gallos, L., Havlin, S., Liljeros, F., Muchnik, L., Stanley, H. & Makse, H. & Dibernardo, M. Identification of influential spreaders in complex networks. *Nature physics*. **6(11)**, 888 (2010)

[6] Lahav, N., Ksherim, B., Ben-Simon, E., Maron-Katz, A., Cohen, R. & Havlin, S. K-shell decomposition reveals hierarchical cortical organization of the human brain. *New Journal of Physics.* **18(8)**, 083013 (2016)

[7] Casey, S., Baylot, V., & Felsher, D. The MYC oncogene is a global regulator of the immune response. *Blood.* **131(18)**, 2007–2015 (2018)

[8] Canon, E., Jouneau, L., Blachère, T., Peynot, N., Daniel, N., Boulanger, L., Maulny, L., Archilla, C., Voisin, S., Jouneau, A. & others. Progressive methylation of POU5F1 regulatory regions during blastocyst development. *Reproduction.* **156(2)**, 145–161 (2018)

[9] Kehler, J., Tolkunova, E., Koschorz, B., Pesce, M., Gentile, L., Boiani, M., Lomelí, H., Nagy, A., McLaughlin, K., Schöler, H. & others. Oct4 is required for primordial germ cell survival. *EMBO reports.* **5(11)**, 1078-1083 (2004)

[10] Pajares, M., Jiménez-Moreno, N., García-Yagüe, A., Escoll, M., de Ceballos, M., Van Leuven, F., Rábano, A., Yamamoto, M., Rojo, A. & Cuadrado, A. Transcription factor NFE2L2/NRF2 is a regulator of macroautophagy genes. *Autophagy.* **12(10)**, 1902–1916 (2016)

[11] Xu, X., Luo, P., Wang, Y., Cui, Y. & Miao, L. Nuclear factor (erythroid-derived 2)-like 2 (NFE2L2) is a novel therapeutic target for diabetic complications. *Journal of international medical research.* **41(1)**, 13-19 (2013)

[12] Thomas, A. CREBBP-mutated cancers HAT-tricked. *Science Translational Medicine.* **7(317)**, 317ec212–317ec212 (2015)

[13] Lodato, M., Ng, C., Wamstad, J., Cheng, A., Thai, K., Fraenkel, E., Jaenisch, R. & Boyer, L. SOX2 co-occupies distal enhancer elements with distinct POU factors in ESCs and NPCs to specify cell state. *PLoS genetics.* **9(2)** (2013)

[14] Cheng, A., Bouchard-Cannon, P., Hegazi, S., Lowden, C., Fung, S., Chiang, C., Ness, R. & Cheng, H. SOX2-dependent transcription in clock neurons promotes the robustness of the central circadian pacemaker. *Cell reports.* **26(12)** 3191–3202 (2019)

[15] Vega, S., Morales, A., Ocaña, O., Valdés, F., Fabregat, I. & Nieto, M. & Nieto, M. Snail blocks the cell cycle and confers resistance to cell death. *Genes & development.* **18(10)** 1131–1143 (2004)

[16] Lim, S., Becker, A., Zimmer, A., Lu, J., Buettner, R. & Kirfel, J. SNAI1-mediated epithelial-mesenchymal transition confers chemoresistance and cellular plasticity by regulating genes involved in cell death and stem cell maintenance. *PloS one.* **8(6)** (2013)

[17] De Craene, B., Denecker, G., Vermassen, P., Taminau, J., Mauch, C., Derore, A., Jonkers, J., Fuchs, E., Berx, G. Epidermal Snail expression drives skin cancer initiation and progression through enhanced cytoprotection, epidermal stem/progenitor cell expansion and enhanced metastatic potential. *Cell Death & Differentiation.* **21(2)** 310–320 (2014)

[18] Horvay, K., Jardé, T., Casagranda, F., Perreau, V., Haigh, K., Nefzger, C., Akhtar, R., Gridley, T., Berx, G., Haigh, J. & others. Snai1 regulates cell lineage allocation and stem cell maintenance in the mouse intestinal epithelium. *The EMBO journal.* **34(10)** 1319–1335 (2015)

[19] Guan, L., Zhu, S., Han, Y., Yang, C., Liu, Y., Qiao, L., Li, X., Li, H. & Lin, J. Knockout of CTNNB1 by CRISPR-Cas9 technology inhibits cell proliferation through the Wnt/β-catenin signaling pathway. *Biotechnology letters.* **40(3)** 501-508 (2018)

[20] Aziz, A., Liu, Q., Dilworth & F. Regulating a master regulator: establishing tissue-specific gene expression in skeletal muscle. *Epigenetics.* **5(8)** 691–695 (2010)

[21] Zhang, S., Li, L., Qiao, H., Yang, X., Chen, L. & Luo, X. Regulation of the Antioxidant Response by MyoD Transcriptional Coactivator in Castration-resistant Prostate Cancer Cells. *Urology.* **123** 296–e9 (2019)

[22] Fukuda, M., Yoshizawa, T., Karim, M., Sobuz, S., Korogi, W., Kobayasi, D., Okanishi, H., Tasaki, M., Ono, K., Sawa, T. & others. SIRT7 has a critical role in bone formation by regulating lysine acylation of SP7/Osterix. *Nature communications.* **9(1)** 1–24 (2018)

[23] Yoshida, C., Komori, H., Maruyama, Z., Miyazaki, T., Kawasaki, K., Furuichi, T., Fukuyama, R., Mori, M., Yamana, K., Nakamura, K. & others. SP7 inhibits osteoblast differentiation at a late stage in mice. *PloS one.* **7(3)** (2012)

[24] Huja, S., Oren, Y., Biran, D., Meyer, S., Dobrindt, U., Bernhard, J., Becher, D., Hecker, M., Sorek, R. & Ron, E. Fur is the master regulator of the extraintestinal pathogenic Escherichia coli response to serum. *Mbio.* **5(4)** e01460–14 (2014)

[25] Seo, S., Kim, D., O'Brien, E., Szubin, R. & Palsson, B. Decoding genome-wide GadEWX-transcriptional regulatory networks reveals multifaceted cellular responses to acid stress in Escherichia coli. *Nature communications.* **6(1)** 1–8 (2015)

[26] Castanié-Cornet, M., Cam, K., Bastiat, B., Cros, A., Bordes, P. & Gutierrez, C. Acid stress response in Escherichia coli: mechanism of regulation of gadA transcription by RcsB and GadE. *Nucleic acids research.* **38(11)** 3546–3554 (2010)

[27] Duval, V. & Lister, I. MarA, SoxS and Rob of Escherichia coli–Global regulators of multidrug resistance, virulence and stress response. *International journal of biotechnology for wellness industries.* **2(3)** 101 (2013)

[28] Raivio, T. Everything old is new again: an update on current research on the Cpx envelope stress response. *Biochimica et Biophysica Acta (BBA)-Molecular Cell Research.* **1843(8)** 1529–1541 (2014)

[29] Prior, H. & Walter, M. SOX genes: architects of development. *Molecular medicine.* **2(4)** 405–412 (1996)

[30] Li, Z. & Demple, B. & Walter, M. SoxS, an activator of superoxide stress genes in Escherichia coli. Purification and interaction with DNA. *Journal of Biological Chemistry.* **269(28)** 18371–18377 (1994)

[31] Kuchinski, K., Brimacombe, C., Westbye, A., Ding, H. & Beatty, J. The SOS response master regulator LexA regulates the gene transfer agent of Rhodobacter capsulatus and represses transcription of the signal transduction protein CckA. *Journal of bacteriology.* **198(7)** 1137–1148 (2016)

[32] Hanlon, S., Rizzo, J., Tatomer, D., Lieb, J. & Buck, M. The stress response factors Yap6, Cin5, Phd1, and Skn7 direct targeting of the conserved co-repressor Tup1-Ssn6 in S. cerevisiae. *PloS one.* **6(4)** e19060 (2011)

[33] Martínez-Montañés, F., Rienzo, A., Poveda-Huertes, D., Pascual-Ahuir, A. & Proft, M. Activator and repressor functions of the Mot3 transcription factor in the osmostress response of Saccharomyces cerevisiae. *Eukaryotic Cell.* **12(5)** 636–647 (2013)

[34] Thomas, A., Belaidi, E., Aron-Wisnewsky, J., Van Der Zon, G., Levy, P., Clement, K., Pepin, J., Godin-Ribuot, D. & Guigas, B. Hypoxia-inducible factor prolyl hydroxylase 1 (PHD1) deficiency promotes hepatic steatosis and liver-specific insulin resistance in mice. *Scientific reports.* **6** 24618 (2016)

[35] Ohno, M., Hiraoka, Y., Matsuoka, T., Tomimoto, H., Takao, K., Miyakawa, T., Oshima, N., Kiyonari, H., Kimura, T., Kita, T. & others. Nardilysin regulates axonal maturation and myelination in the central and peripheral nervous system. *Nature neuroscience.* **12(12)** 1506 (2009)

[36] Kuang, Z., Ji, H., Boeke, J. Stress response factors drive regrowth of quiescent cells. *Current genetics.* **64(4)** 807–810 (2018)

[37] Shi, W., Chen, Z., Li, L., Liu, H., Zhang, R., Cheng, Q., Xu, D. & Wu, L. Unravel the molecular mechanism of XBP1 in regulating the biology of cancer cells. *Journal of Cancer.* **10(9)** 2035 (2019)

[38] Vázquez-Marín, J., Gutiérrez-Triana, J., Almuedo-Castillo, M., Buono, L., Gómez-Skarmeta, J., and Mateo, J.,Wittbrodt, J. & Martínez-Morales, J. yap1b, a divergent Yap/Taz family member, cooperates with yap1 in survival and morphogenesis via common transcriptional targets. *Development.* **146(13)** dev173286 (2019)

[39] Lee, J., Godon, C., Lagniel, G., Spector, D., Garin, J., Labarre, J. & Toledano, M. Yap1 and Skn7 control two specialized oxidative stress response regulons in yeast. *Journal of Biological Chemistry.* **274(23)** 16040–16046 (1999)

[40] Nomura, W., Maeta, K., Kita, K., Izawa, S. & Inoue, Y. Role of Gcn4 for adaptation to methylglyoxal in Saccharomyces cerevisiae: Methylglyoxal attenuates protein synthesis through phosphorylation of eIF2α. *Biochemical and biophysical research communications.* **376(4)** 738–742 (2008)
